# Supplementary figures and images for: Assessing the replicability of spatial gene expression using atlas data from the adult mouse brain
Source: PLoS Biol. 2021 Jul 19;19(7):e3001341. doi: 10.1371/journal.pbio.3001341 (PMC8321401; doi:10.1371/journal.pbio.3001341)

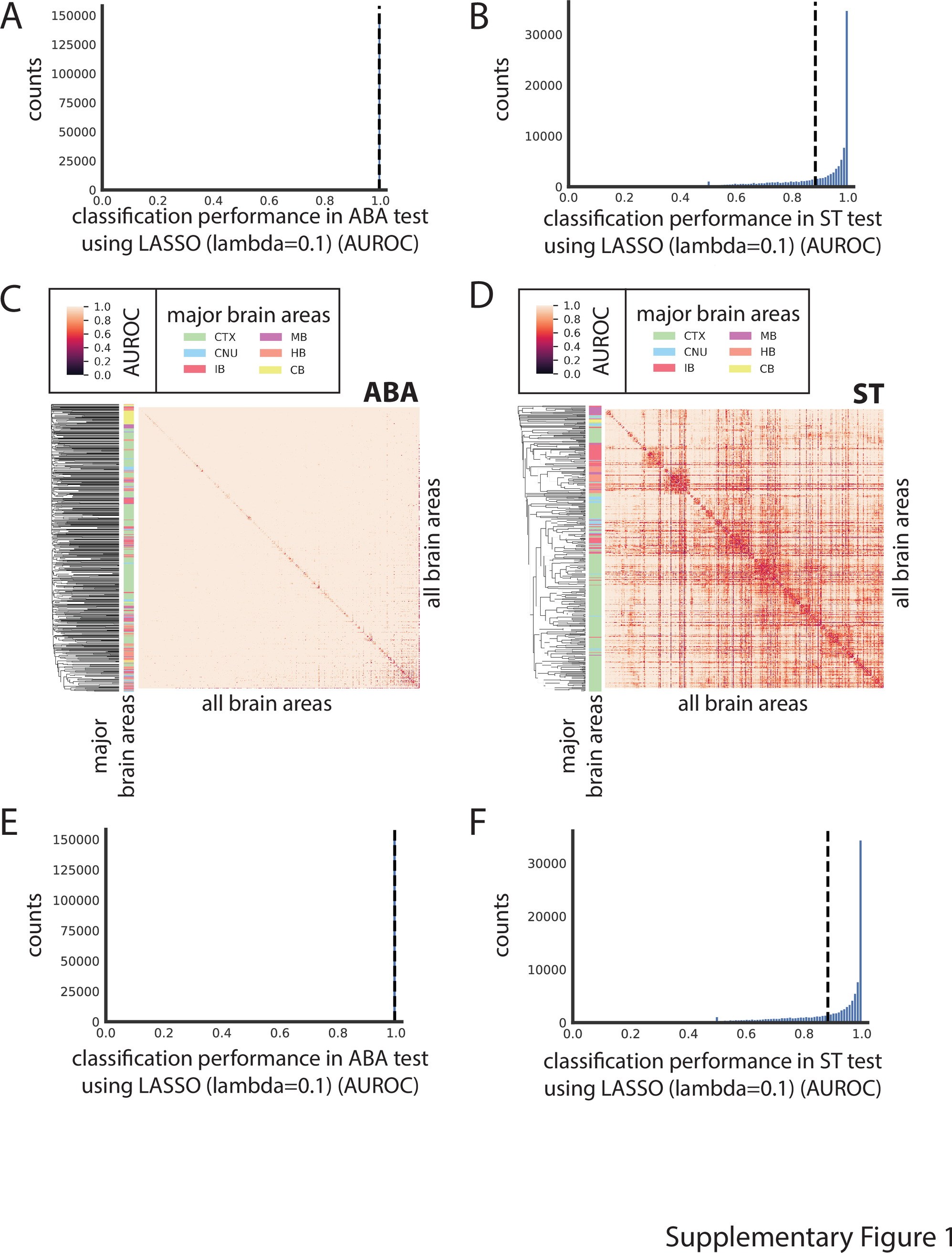

Supplement: S1 Fig — Histogram of classification performance of LASSO (lambda = 0.1) in (A) ABA test fold and (B) ST. (A) and (B) represent the upper triangular of Fig 2A and Fig 2B, respectively. Black dashed vertical line represents the mean. Heat map of AUROC for classifying leaf brain areas from all other leaf brain areas in (C) ABA and (D) ST using LASSO (lambda = 0.1) using a different random train/test split with a seed = 9 relative to Fig 2A and 2B. Dendrograms on the far left side represent clustering of leaf brain areas based on the inverse of AUROC; areas with an AUROC near 0.5 get clustered together, while areas with an AUROC near 1 are further apart. Color bar on the left represents the major brain structure that the leaf brain area is grouped under. These areas include CTX, MB, CB, CNU, HB, and IB. Histogram of classification performance of LASSO (lambda = 0.1) in (E) ABA test fold and (F) ST. (E) and (F) represent the upper triangular of (C) and (D), respectively. Black dashed vertical line represents the mean. ABA, Allen Brain Atlas; AUROC, area under the receiver operating curve; CB, cerebellum; CNU, striatum and pallidum; CTX, cortex; HB, hindbrain; IB, thalamus and hypothalamus; LASSO, least absolute shrinkage and selection operator; MB, midbrain; ST, spatial transcriptomics. (TIF) [file pbio.3001341.s001.tif]

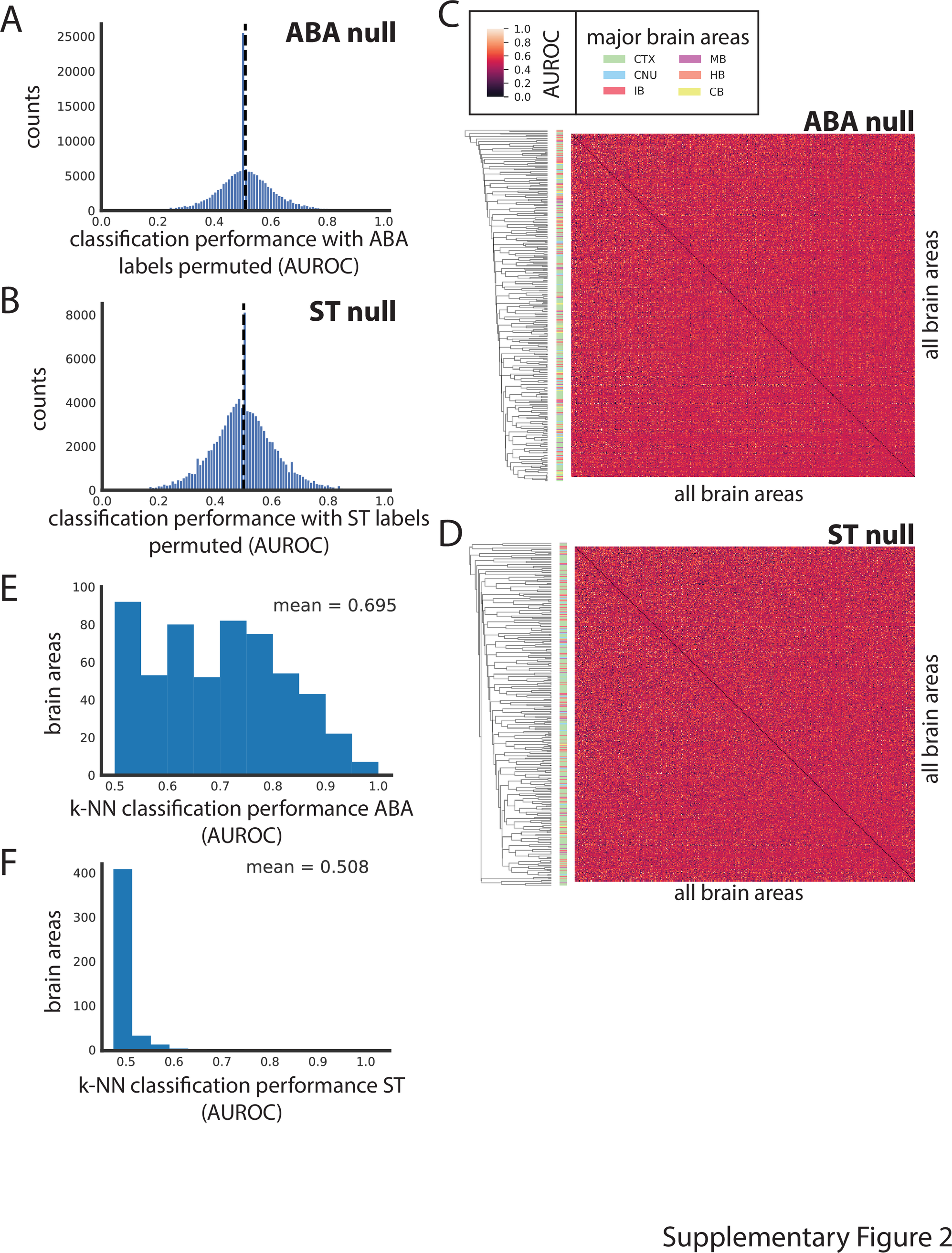

Supplement: S2 Fig — Upper triangular (A) histogram and (C) heat map of AUROC for classifying leaf brain areas from all other leaf brain areas in ABA using LASSO (lambda = 0.1) when brain area labels are randomly permuted. (B) and (D) same as (A) and (C), respectively, but for ST with labels permuted. For heat maps (C, D), dendrograms on the far left side represent clustering of leaf brain areas based on the inverse of AUROC; areas with an AUROC near 0.5 get clustered together, while areas with an AUROC near 1 are further apart. Color bar on the left represents the major brain structure that the leaf brain area is grouped under. These areas include CTX, MB, CB, CNU, HB, and IB. Distribution of performance (AUROC) of classifying each brain area using k-NN, a multiclass classifier, with default parameters (k = 5) for (E) ABA and (F) ST. Mean AUROC is shown in the upper left of each plot. ABA, Allen Brain Atlas; AUROC, area under the receiver operating curve; CB, cerebellum; CNU, striatum and pallidum; CTX, cortex; HB, hindbrain; IB, thalamus and hypothalamus; k-NN, k-nearest neighbors; LASSO, least absolute shrinkage and selection operator; MB, midbrain; ST, spatial transcriptomics. (TIF) [file pbio.3001341.s002.tif]

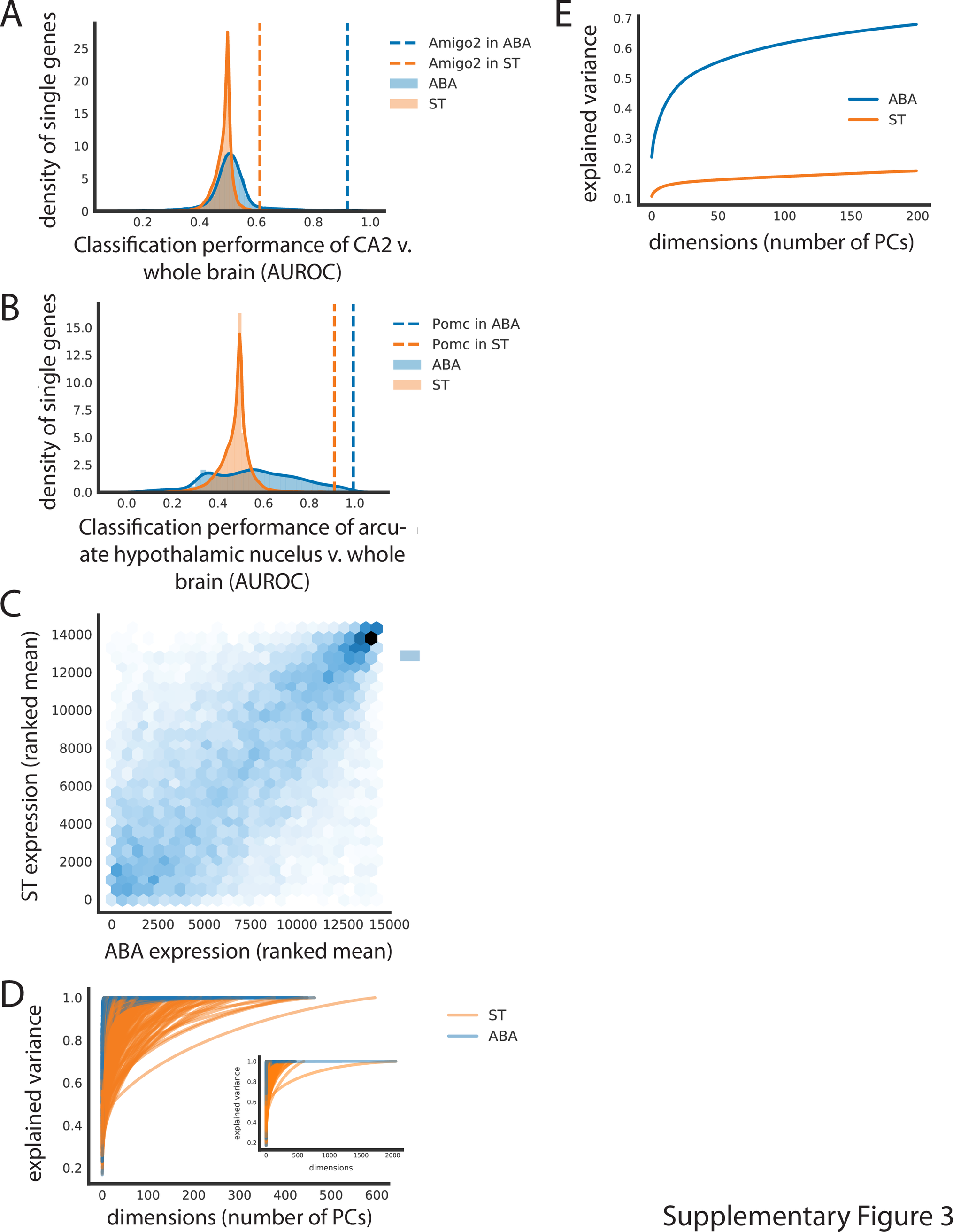

Supplement: S3 Fig — Distribution of classifying (A) CA2 and (B) arcuate hypothalamic nucleus against the rest of the brain using single genes. Distributions of all single genes shown for classification in ABA (blue) and ST (orange). Dashed lines represent the marker gene (A) Amigo2 or (B) Pomc for classification in ABA (blue) or ST (orange). (C) Relative expression between the ST and ABA datasets as a density plot. Expression is plotted as the ranked mean for each gene across all samples. (D) Cumulative explained variance curves for PCA in ST (orange) and ABA (blue). Each curve represents one leaf brain area. The total number of principal components per brain area is equal to the number of samples in that area. ABA areas that had more samples than ST are randomly down-sampled accordingly. For both datasets, the Caudoputamen, the largest region, is removed to allow visualization; full figure shown in inset plot. (E) Cumulative explained variance curves for 200 PCs in the whole ST (orange) and whole ABA (blue) datasets. ABA, Allen Brain Atlas; AUROC, area under the receiver operating curve; PCA, principal component analysis; ST, spatial transcriptomics. (TIF) [file pbio.3001341.s003.tif]

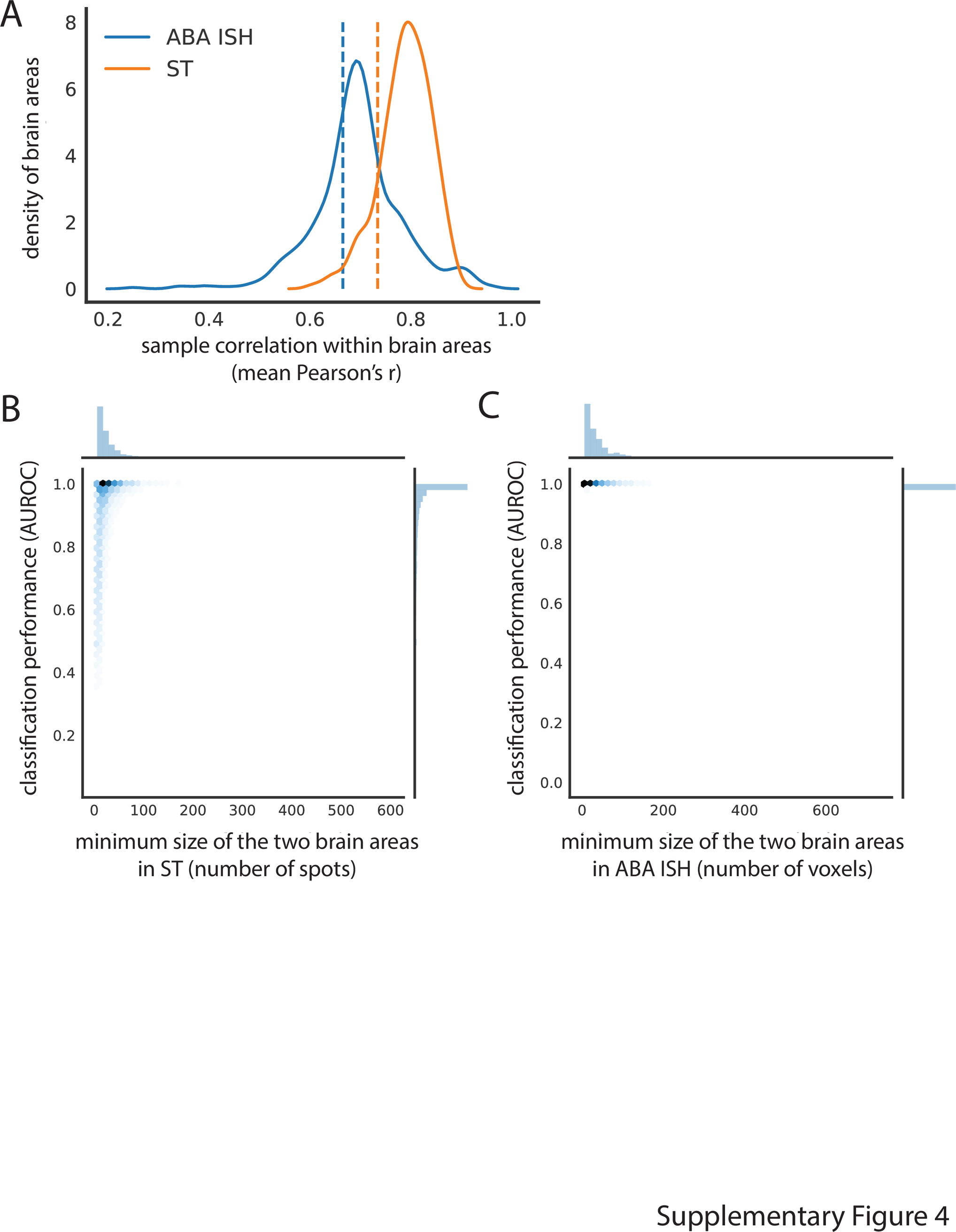

Supplement: S4 Fig — (A) Distribution of sample correlation within each of the leaf brain areas for ABA (blue) and ST (orange). Vertical dashed line represents the mean for the correspondingly colored distribution. Test set AUROC in (B) ST and (C) ABA as a function of the number of samples per brain area. The minimum of the 2 brain areas involved in classification is shown. ABA, Allen Brain Atlas; AUROC, area under the receiver operating curve; ISH, in situ hybridization; ST, spatial transcriptomics. (TIF) [file pbio.3001341.s004.tif]

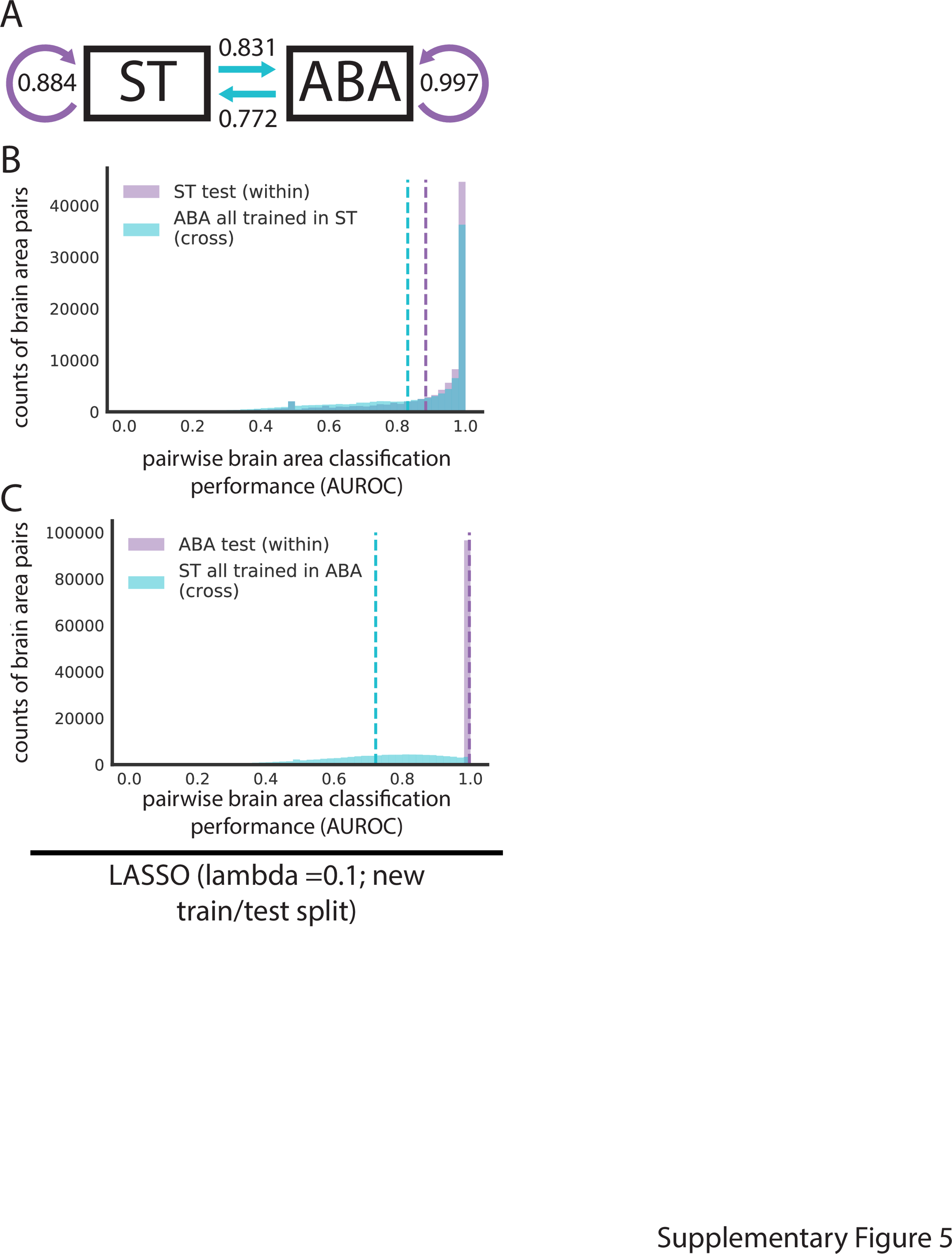

Supplement: S5 Fig — (A) Models trained with overlapping genes and brain areas between ST and ABA datasets are evaluated within dataset on the test fold and across dataset on the entire opposite dataset as illustrated in Fig 1C. Summary diagrams showing mean AUROC for within-dataset test set performance (purple arrow) and cross-dataset performance with models trained in the opposite dataset (light blue arrow) for (A) LASSO (lambda = 0.1) with a new random train/test split (seed = 9) relative to Fig 3A–3C. Distributions of AUROCs for within- (purple) and cross-dataset (light blue) performance for (B) LASSO (lambda = 0.1) trained in ST with new train/test split and (C) LASSO (lambda = 0.1) trained in ABA with new train/test split. In both plots, dashed vertical lines represent the mean of the corresponding colored distribution. ABA, Allen Brain Atlas; AUROC, area under the receiver operating curve; LASSO, least absolute shrinkage and selection operator; ST, spatial transcriptomics. (TIF) [file pbio.3001341.s005.tif]

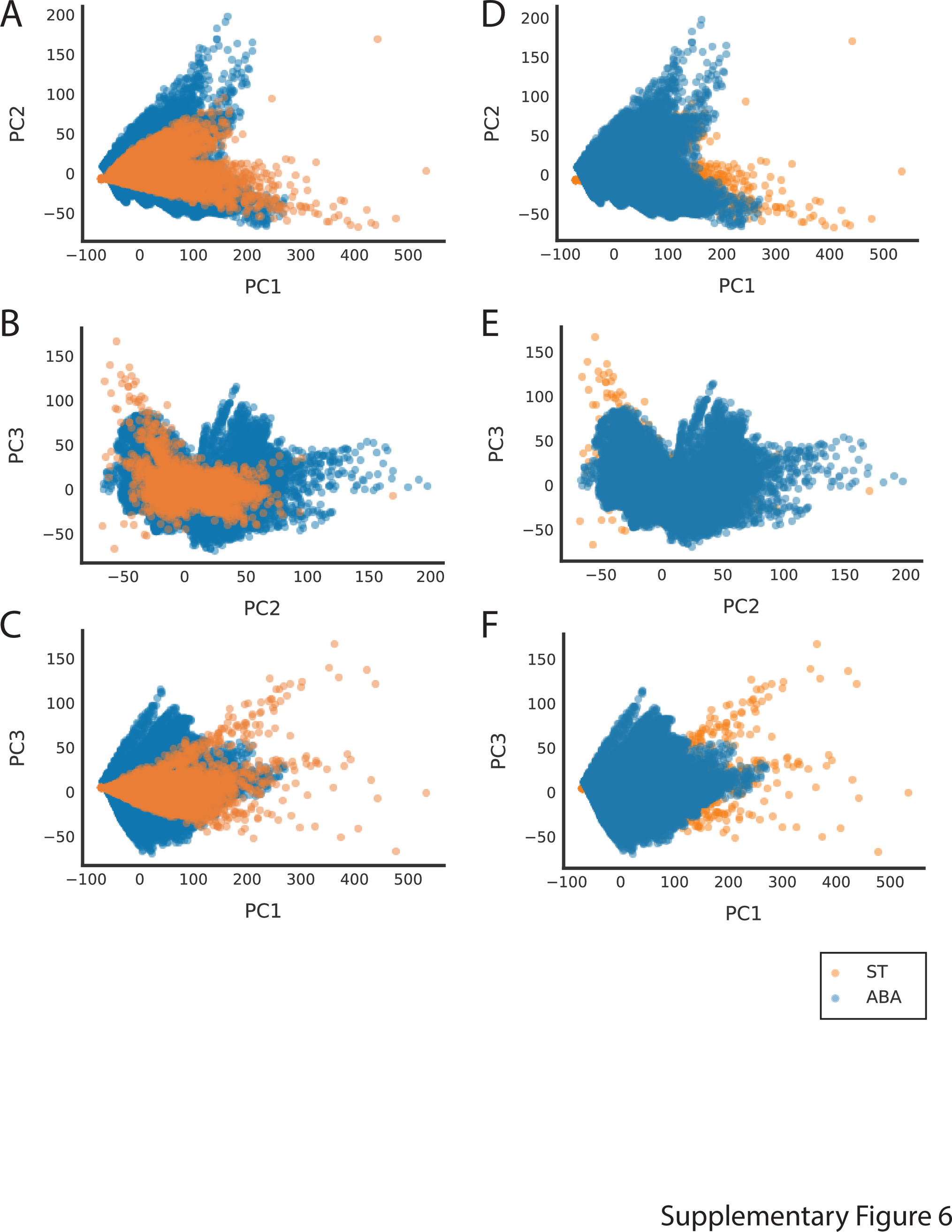

Supplement: S6 Fig — Plots showing ABA (blue) and ST (orange) datasets visualized together in low-dimensional space after dimensionality reduction with PCA. (A–C) show ST plotted on top of ABA, while (D–F) show the same PCs with ABA plotted on top of ST. Plots show (A, D) PC1 vs. PC2, (B, E) PC2 vs. PC3, and (C, F) PC1 vs. PC3. ABA, Allen Brain Atlas; PCA, principal component analysis; ST, spatial transcriptomics. (TIF) [file pbio.3001341.s006.tif]

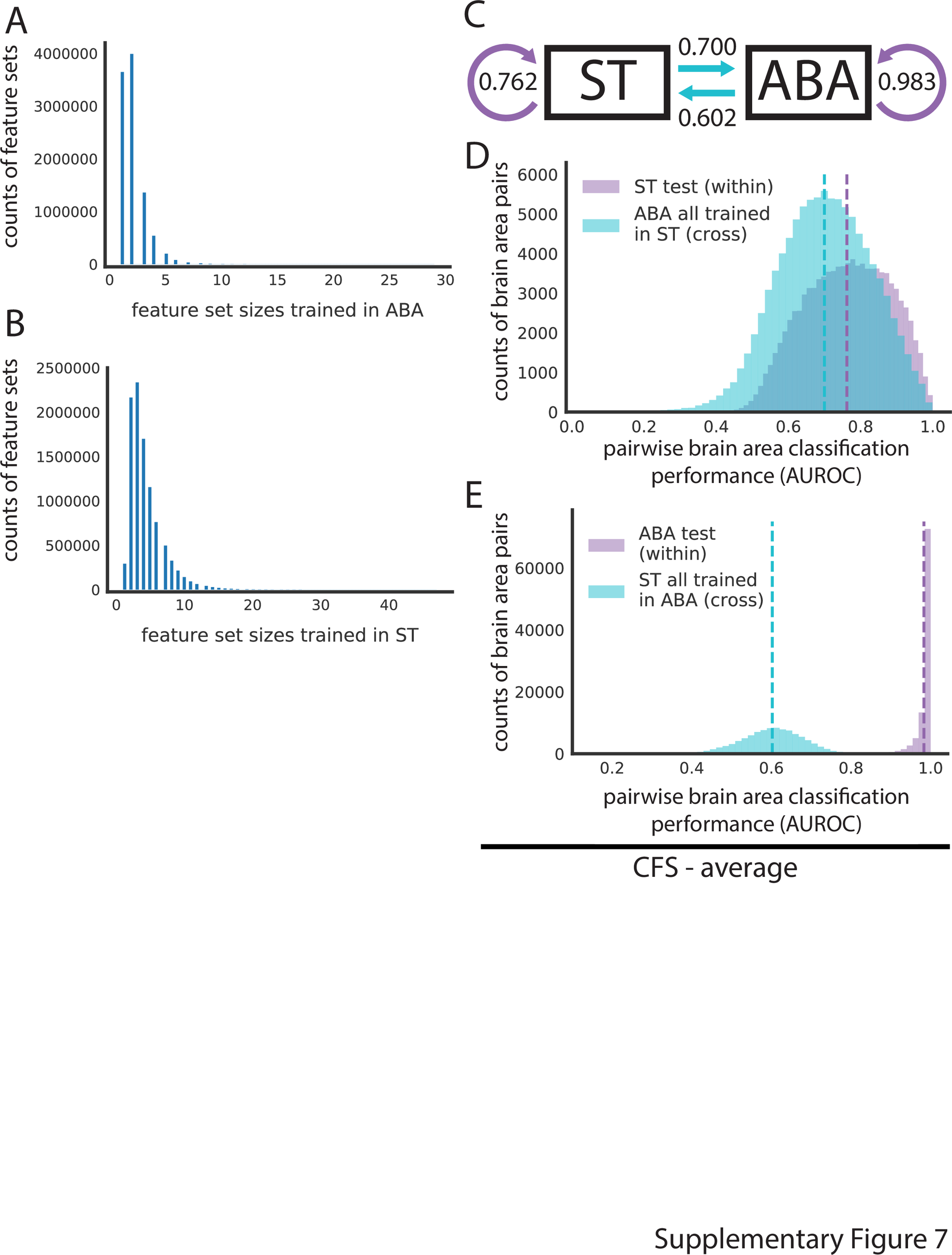

Supplement: S7 Fig — Distribution of CFS feature set sizes for (A) ABA and (B) ST. Models trained with overlapping genes and brain areas between ST and ABA datasets are evaluated within dataset on the test fold and across dataset on the entire opposite dataset as illustrated in Fig 1C. (C) Summary diagrams showing mean AUROC for within-dataset test set performance (purple arrow) and cross-dataset performance with models trained in the opposite dataset (light blue arrow) using the average of 100 feature sets chosen with CFS. Distributions of AUROCs for within- (purple) and cross-dataset (light blue) performance for 100 averaged CFS picked gene sets trained (D) in ST and (E) in ABA. In both plots, dashed vertical lines represent the mean of the correspondingly colored distribution. ABA, Allen Brain Atlas; AUROC, area under the receiver operating curve; CFS, correlation-based feature selection; ST, spatial transcriptomics. (TIF) [file pbio.3001341.s007.tif]

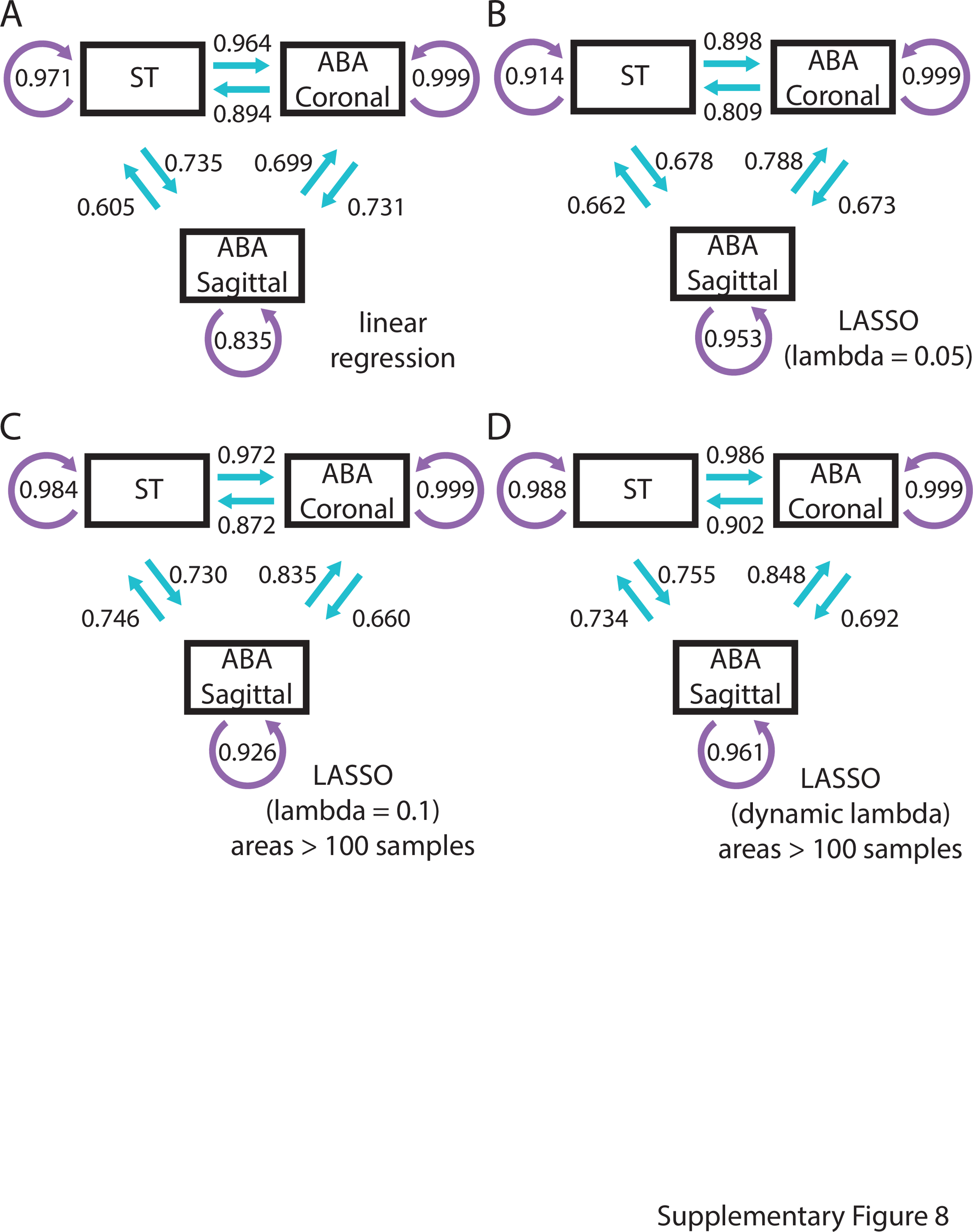

Supplement: S8 Fig — Separating out the 2 planes of slicing in the ABA and treating them alongside the ST dataset as 3 different datasets for cross-dataset learning. Summary diagram showing mean AUROCs using (A) linear regression and (B) LASSO (lambda = 0.05). (C, D) Same cross-dataset analysis as (A, B) but looking only at brain areas with at least 100 samples for (C) fixed lambda = 0.1 and (D) dynamically fitted lambda for each brain area pair. In all 4 summary diagrams, cross-dataset arrows originate from the dataset that the model is trained in and point to the dataset that those models are tested in. ABA, Allen Brain Atlas; AUROC, area under the receiver operating curve; LASSO, least absolute shrinkage and selection operator; ST, spatial transcriptomics. (TIF) [file pbio.3001341.s008.tif]

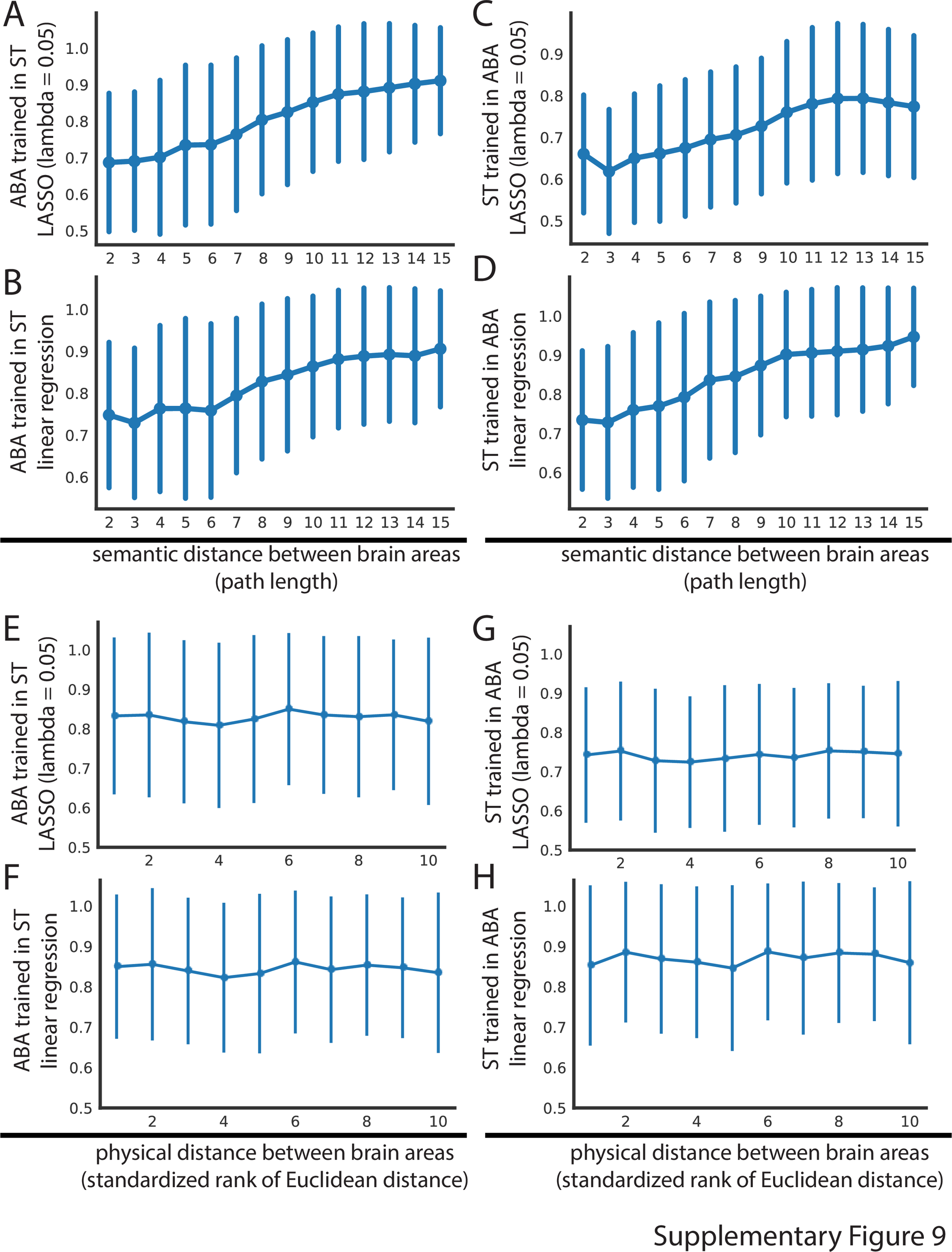

Supplement: S9 Fig — Cross-dataset AUROCs (x-axis) of classifying all leaf brain areas from all other leaf brain areas for (A) ABA using LASSO (lambda = 0.05) trained in ST, (B) ABA using linear regression trained in ST, (C) ST using LASSO (lambda = 0.05) trained in ABA, and (D) ST using linear regression trained in ABA as a function of path length (x-axis) in the ARA naming hierarchy between the 2 brain areas being classified. The same AUROCs (y-axis) from (A–D) shown in (E–H), respectively, as a function of minimum Euclidean distance between the 2 brain areas in the ARA (x-axis). Euclidean distance on the x-axis is binned into deciles for visualization. All plots show mean AUROCs (points) with standard deviation (vertical bars). ABA, Allen Brain Atlas; ARA, Allen Reference Atlas; AUROC, area under the receiver operating curve; LASSO, least absolute shrinkage and selection operator; ST, spatial transcriptomics. (TIF) [file pbio.3001341.s009.tif]

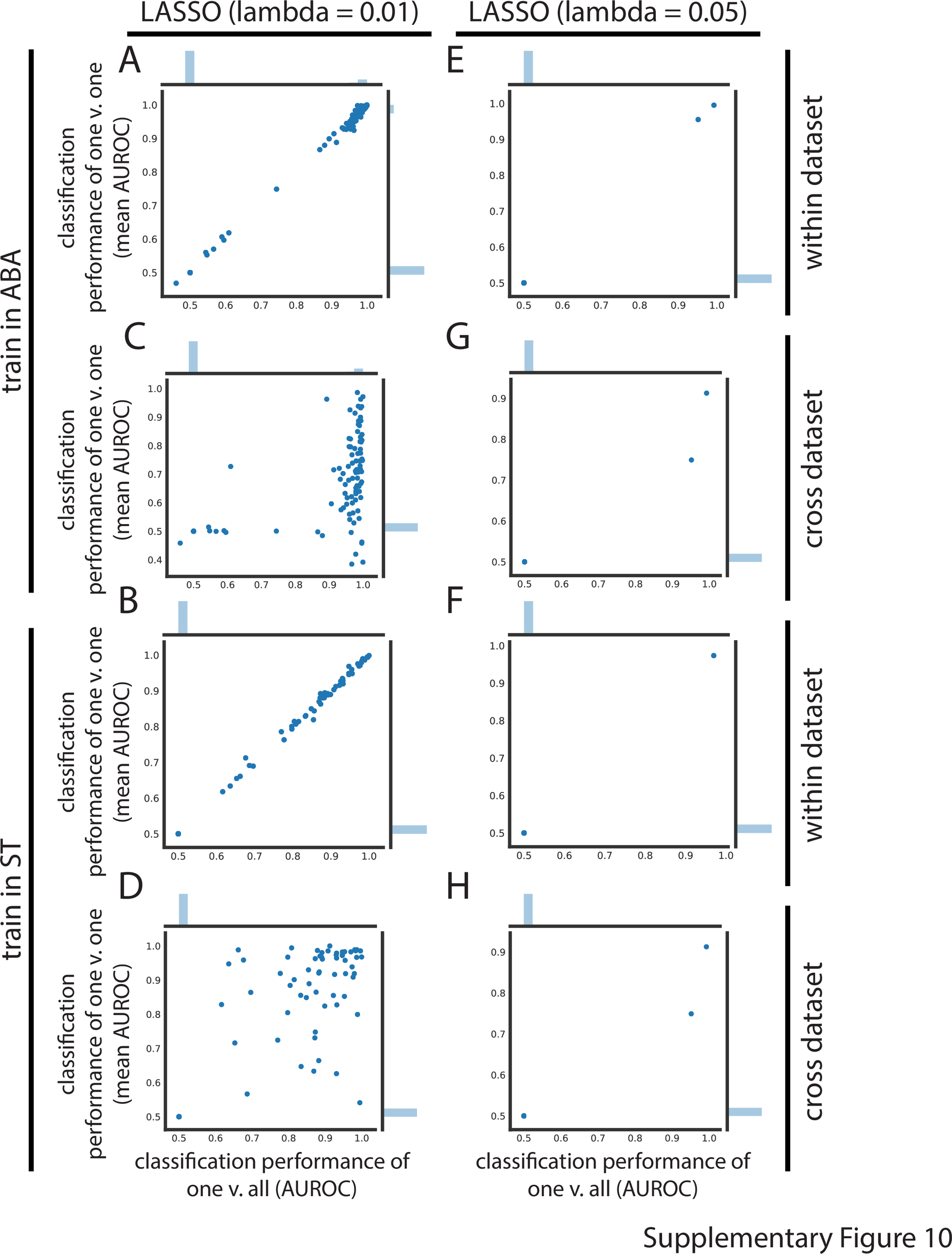

Supplement: S10 Fig — LASSO (lambda = 0.01) one vs. all test set performance (x-axis) vs. average one vs. one performance (y-axis) of the same dataset (A) in ABA and (B) in ST. (C, D) Same as (A and B), but one vs. one performance (y-axis) is accessed in the opposite dataset for (C) train one vs. all in ABA and test one vs. one in ST and (D) train one vs. all in ST and test one vs. one in ABA. (E–H) Same as (A–D), respectively, but using LASSO (lambda = 0.05). ABA, Allen Brain Atlas; AUROC, area under the receiver operating curve; LASSO, least absolute shrinkage and selection operator; ST, spatial transcriptomics. (TIF) [file pbio.3001341.s010.tif]

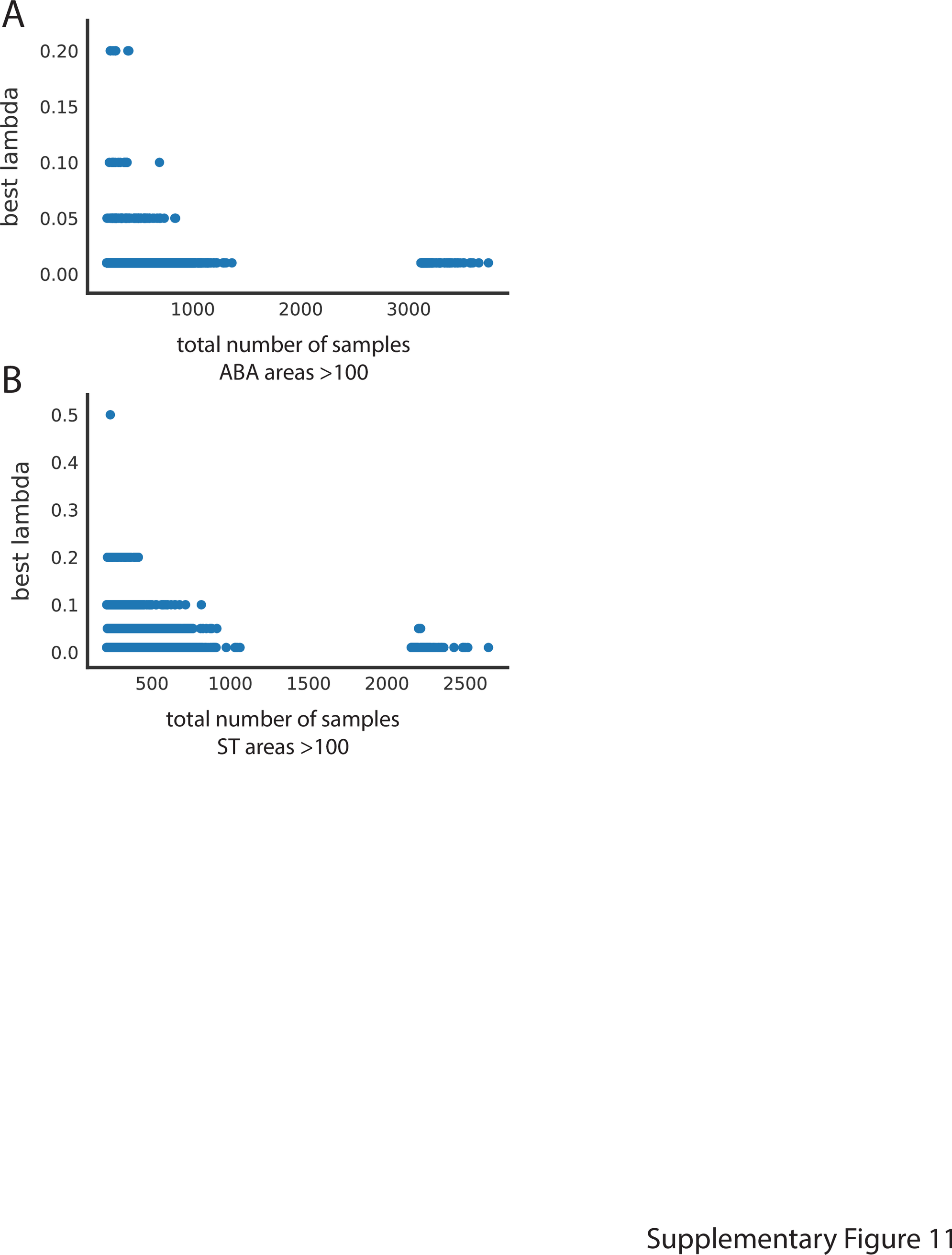

Supplement: S11 Fig — Plots showing the best lambda for LASSO when dynamically fit across various possible values as a function of brain area sample size in (A) ABA and (B) ST. ABA, Allen Brain Atlas; LASSO, least absolute shrinkage and selection operator; ST, spatial transcriptomics. (TIF) [file pbio.3001341.s011.tif]
