# Supplementary material for: Assessing the replicability of spatial gene expression using atlas data from the adult mouse brain
Source: PLoS Biol. 2021 Jul 19;19(7):e3001341. doi: 10.1371/journal.pbio.3001341 (PMC8321401; doi:10.1371/journal.pbio.3001341)
Supplement: S1 Table — ABA data are randomly down-sampled here to have the same sample size as ST. Note that the not-batch corrected case is not down-sampled, but it is filtered for the brain areas that are included in the batch corrected mean AUROCs after down-sampling. ABA, Allen Brain Atlas; AUROC, area under the receiver operating curve; ST, spatial transcriptomics. (PDF) [file pbio.3001341.s012.pdf]

Supplementary Table 1

Cross-Dataset Classification Performance (mean AUROC)

	ST to ABA		ABA to ST	
	within test	cross	within test	cross
Batch Corrected	0.897	0.838	0.991	0.715
Not Batch Corected	0.897	0.839	0.998	0.734
