# Supplementary material for: Assessing the replicability of spatial gene expression using atlas data from the adult mouse brain
Source: PLoS Biol. 2021 Jul 19;19(7):e3001341. doi: 10.1371/journal.pbio.3001341 (PMC8321401; doi:10.1371/journal.pbio.3001341)
Supplement: S2 Table — ABA, Allen Brain Atlas; AUROC, area under the receiver operating curve; LASSO, least absolute shrinkage and selection operator; ST, spatial transcriptomics. (PDF) [file pbio.3001341.s013.pdf]

Supplementary Table 2

ST to ABA LASSO, alpha=0.1 auroc file = "STtoABA_ABAall_f1_0p1_051420.csv"

AUROC = 1; path length = 2

Brain Area 1				Brain Area 2			
id	acronym	name	parent	id	acronym	name	parent
9	SSp-tr6a	Primary somatosensory area, trunk, layer 6a	361	1086	SSp-tr4	Primary somatosensory area, trunk, layer 4	361
9	SSp-tr6a	Primary somatosensory area, trunk, layer 6a	361	670	SSp-tr2/3	Primary somatosensory area, trunk, layer 2/3	361
1005	AUDp6b	Primary auditory area, layer 6b	1002	735	AUDp1	Primary auditory area, layer 1	1002
1102	SSp-m6a	Primary somatosensory area, mouth, layer 6a	345	950	SSp-m4	Primary somatosensory area, mouth, layer 4	345
1066	VISam2/3	Anteromedial visual area, layer 2/3	394	1046	VISam6a	Anteromedial visual area, layer 6a	394
308	PTLp6a	Posterior parietal association areas, layer 6a	22	241	PTLp2/3	Posterior parietal association areas, layer 2/3	22
1030	SSp-ll1	Primary somatosensory area, lower limb, layer 1	337	478	SSp-ll6a	Primary somatosensory area, lower limb, layer 6a	337
905	VISal2/3	Anterolateral visual area, layer 2/3	402	601	VISal6a	Anterolateral visual area, layer 6a	402
600	AUDd2/3	Dorsal auditory area, layer 2/3	1011	156	AUDd6a	Dorsal auditory area, layer 6a	1011
251	AUDp2/3	Primary auditory area, layer 2/3	1002	847	AUDp5	Primary auditory area, layer 5	1002
583	CLA	Claustrum	703	780	PA	Posterior amygdalar nucleus	703
1035	SSs4	Supplemental somatosensory area, layer 4	378	862	SSs6a	Supplemental somatosensory area, layer 6a	378
729	TEa6a	Temporal association areas, layer 6a	541	97	TEa1	Temporal association areas, layer 1	541
478	SSp-ll6a	Primary somatosensory area, lower limb, layer 6a	337	113	SSp-ll2/3	Primary somatosensory area, lower limb, layer 2/3	337
816	AUDp4	Primary auditory area, layer 4	1002	954	AUDp6a	Primary auditory area, layer 6a	1002

AUROC <= 0.5; path length = 2

Brain Area 1				Brain Area 2			
id	acronym	name	parent	id	acronym	name	parent
1114	VISal4	Anterolateral visual area, layer 4	402	1074	VISal1	Anterolateral visual area, layer 1	402
606	RSPv2	Retrosplenial area, ventral part, layer 2	886	622	RSPv6b	Retrosplenial area, ventral part, layer 6b	886
472	MEApd-a	Medial amygdalar nucleus, posterodorsal part, sublayer a	426	480	MEApd-b	Medial amygdalar nucleus, posterodorsal part, sublayer b	426
1072	MGd	Medial geniculate complex, dorsal part	475	1088	MGm	Medial geniculate complex, medial part	475
1088	MGm	Medial geniculate complex, medial part	475	1079	MGv	Medial geniculate complex, ventral part	475
980	PMd	Dorsal premammillary nucleus	467	1004	PMv	Ventral premammillary nucleus	467
559	CEAm	Central amygdalar nucleus, medial part	536	544	CEAc	Central amygdalar nucleus, capsular part	536
281	VISam1	Anteromedial visual area, layer 1	394	1066	VISam2/3	Anteromedial visual area, layer 2/3	394
1042	TTd2	Taenia tecta, dorsal part, layer 2	597	1050	TTd3	Taenia tecta, dorsal part, layer 3	597
148	GU4	Gustatory areas, layer 4	1057	187	GU5	Gustatory areas, layer 5	1057
148	GU4	Gustatory areas, layer 4	1057	662	GU6b	Gustatory areas, layer 6b	1057
783	AId6a	Agranular insular area, dorsal part, layer 6a	104	1101	AId5	Agranular insular area, dorsal part, layer 5	104
381	SNr	Substantia nigra, reticular part	323	616	CUN	Cuneiform nucleus	323
74	VISl6a	Lateral visual area, layer 6a	409	973	VISl2/3	Lateral visual area, layer 2/3	409
74	VISl6a	Lateral visual area, layer 6a	409	421	VISl1	Lateral visual area, layer 1	409
416	PAA2	Piriform-amygdalar area, pyramidal layer	788	424	PAA3	Piriform-amygdalar area, polymorph layer	788
868	PBld	Parabrachial nucleus, lateral division, dorsal lateral part	881	891	PBlv	Parabrachial nucleus, lateral division, ventral lateral part	881
1106	VISC2/3	Visceral area, layer 2/3	677	897	VISC1	Visceral area, layer 1	677
194	LHA	Lateral hypothalamic area	290	173	RCH	Retrochiasmatic area	290
194	LHA	Lateral hypothalamic area	290	226	LPO	Lateral preoptic area	290
194	LHA	Lateral hypothalamic area	290	364	PSTN	Parasubthalamic nucleus	290
368	PERI6b	Perirhinal area, layer 6b	922	692	PERI5	Perirhinal area, layer 5	922
368	PERI6b	Perirhinal area, layer 6b	922	888	PERI2/3	Perirhinal area, layer 2/3	922
1102	SSp-m6a	Primary somatosensory area, mouth, layer 6a	345	2	SSp-m6b	Primary somatosensory area, mouth, layer 6b	345
969	ORBvl1	Orbital area, ventrolateral part, layer 1	746	608	ORBvl6a	Orbital area, ventrolateral part, layer 6a	746
401	VISam4	Anteromedial visual area, layer 4	394	1046	VISam6a	Anteromedial visual area, layer 6a	394
401	VISam4	Anteromedial visual area, layer 4	394	441	VISam6b	Anteromedial visual area, layer 6b	394
540	PERI1	Perirhinal area, layer 1	922	692	PERI5	Perirhinal area, layer 5	922
540	PERI1	Perirhinal area, layer 1	922	888	PERI2/3	Perirhinal area, layer 2/3	922
189	RH	Rhomboid nucleus	51	599	CM	Central medial nucleus of the thalamus	51
880	DTN	Dorsal tegmental nucleus	987	898	PCG	Pontine central gray	987
1066	VISam2/3	Anteromedial visual area, layer 2/3	394	441	VISam6b	Anteromedial visual area, layer 6b	394
255	AV	Anteroventral nucleus of thalamus	239	1113	IAD	Interanterodorsal nucleus of the thalamus	239
965	RSPagl2/3	Retrosplenial area, lateral agranular part, layer 2/3	894	774	RSPagl5	Retrosplenial area, lateral agranular part, layer 5	894
167	AONd	Anterior olfactory nucleus, dorsal part	159	160	AON1	Anterior olfactory nucleus, layer 1	159
167	AONd	Anterior olfactory nucleus, dorsal part	159	183	AONl	Anterior olfactory nucleus, lateral part	159
308	PTLp6a	Posterior parietal association areas, layer 6a	22	340	PTLp6b	Posterior parietal association areas, layer 6b	22
272	AVPV	Anteroventral periventricular nucleus	141	286	SCH	Suprachiasmatic nucleus	141
272	AVPV	Anteroventral periventricular nucleus	141	523	MPO	Medial preoptic area	141
1081	ILA6b	Infralimbic area, layer 6b	44	707	ILA1	Infralimbic area, layer 1	44
1081	ILA6b	Infralimbic area, layer 6b	44	827	ILA5	Infralimbic area, layer 5	44
1081	ILA6b	Infralimbic area, layer 6b	44	556	ILA2/3	Infralimbic area, layer 2/3	44
263	AVP	Anteroventral preoptic nucleus	141	126	PVp	Periventricular hypothalamic nucleus, posterior part	141
1093	PRNc	Pontine reticular nucleus, caudal part	987	534	SUT	Supratrigeminal nucleus	987
240	COApm1	Cortical amygdalar area, posterior part, medial zone, layer 1	663	248	COApm2	Cortical amygdalar area, posterior part, medial zone, layer 2	663
687	RSPv5	Retrosplenial area, ventral part, layer 5	886	622	RSPv6b	Retrosplenial area, ventral part, layer 6b	886
139	ENTl5	Entorhinal area, lateral part, layer 5	918	92	ENTl4	Entorhinal area, lateral part, layer 4	918
1030	SSp-ll1	Primary somatosensory area, lower limb, layer 1	337	113	SSp-ll2/3	Primary somatosensory area, lower limb, layer 2/3	337
574	TRN	Tegmental reticular nucleus	987	534	SUT	Supratrigeminal nucleus	987
837	SUBd-sr	Subiculum, dorsal part, stratum radiatum	509	845	SUBd-sp	Subiculum, dorsal part, pyramidal layer	509
935	ACAd1	Anterior cingulate area, dorsal part, layer 1	39	211	ACAd2/3	Anterior cingulate area, dorsal part, layer 2/3	39
897	VISC1	Visceral area, layer 1	677	857	VISC6a	Visceral area, layer 6a	677

575	CL	Central lateral nucleus of the thalamus	51	599	CM	Central medial nucleus of the thalamus	51
1074	VISal1	Anterolateral visual area, layer 1	402	905	VISal2/3	Anterolateral visual area, layer 2/3	402
1074	VISal1	Anterolateral visual area, layer 1	402	233	VISal5	Anterolateral visual area, layer 5	402
544	CEAc	Central amygdalar nucleus, capsular part	536	551	CEAl	Central amygdalar nucleus, lateral part	536
431	CA2slm	Field CA2, stratum lacunosum-moleculare	423	454	CA2sr	Field CA2, stratum radiatum	423
303	BLAa	Basolateral amygdalar nucleus, anterior part	295	451	BLAv	Basolateral amygdalar nucleus, ventral part	295
266	LSv	Lateral septal nucleus, ventral part	242	258	LSr	Lateral septal nucleus, rostral (rostroventral) part	242
527	AUDd1	Dorsal auditory area, layer 1	1011	600	AUDd2/3	Dorsal auditory area, layer 2/3	1011
646	DP5	Dorsal peduncular area, layer 5	814	496	DP1	Dorsal peduncular area, layer 1	814
118	PVi	Periventricular hypothalamic nucleus, intermediate part	157	223	ARH	Arcuate hypothalamic nucleus	157
616	CUN	Cuneiform nucleus	323	214	RN	Red nucleus	323
772	ACAv5	Anterior cingulate area, ventral part, layer 5	48	810	ACAv6a	Anterior cingulate area, ventral part, 6a	48
269	VISpl2/3	Posterolateral visual area, layer 2/3	425	377	VISpl6a	Posterolateral visual area, layer 6a	425
269	VISpl2/3	Posterolateral visual area, layer 2/3	425	902	VISpl5	Posterolateral visual area, layer 5	425
486	CA3so	Field CA3, stratum oriens	463	471	CA3slm	Field CA3, stratum lacunosum-moleculare	463
1075	TTv2	Taenia tecta, ventral part, layer 2	605	1082	TTv3	Taenia tecta, ventral part, layer 3	605
501	VISpm4	posteromedial visual area, layer 4	533	257	VISpm6a	posteromedial visual area, layer 6a	533
92	ENTl4	Entorhinal area, lateral part, layer 4	918	999	ENTl2/3	Entorhinal area, lateral part, layer 2/3	918
617	MDc	Mediodorsal nucleus of the thalamus, central part	362	636	MDm	Mediodorsal nucleus of the thalamus, medial part	362
1045	ECT6b	Ectorhinal area/Layer 6b	895	977	ECT6a	Ectorhinal area/Layer 6a	895
28	ENTl6a	Entorhinal area, lateral part, layer 6a	918	60	ENTl6b	Entorhinal area, lateral part, layer 6b	918
243	AUDd6b	Dorsal auditory area, layer 6b	1011	156	AUDd6a	Dorsal auditory area, layer 6a	1011
52	ENTl3	Entorhinal area, lateral part, layer 3	918	999	ENTl2/3	Entorhinal area, lateral part, layer 2/3	918
1142	TR3	Postpiriform transition area, layers 3	566	1141	TR2	Postpiriform transition area, layers 2	566
10694	PAR2	Parasubiculum, layer 2	843	10695	PAR3	Parasubiculum, layer 3	843
712	ENTm4	Entorhinal area, medial part, dorsal zone, layer 4	926	664	ENTm3	Entorhinal area, medial part, dorsal zone, layer 3	926
712	ENTm4	Entorhinal area, medial part, dorsal zone, layer 4	926	727	ENTm5	Entorhinal area, medial part, dorsal zone, layer 5	926
565	VISpm5	posteromedial visual area, layer 5	533	257	VISpm6a	posteromedial visual area, layer 6a	533
883	PBls	Parabrachial nucleus, lateral division, superior lateral part	881	891	PBlv	Parabrachial nucleus, lateral division, ventral lateral part	881
1026	SSp-ul6b	Primary somatosensory area, upper limb, layer 6b	369	945	SSp-ul6a	Primary somatosensory area, upper limb, layer 6a	369
471	CA3slm	Field CA3, stratum lacunosum-moleculare	463	479	CA3slu	Field CA3, stratum lucidum	463
471	CA3slm	Field CA3, stratum lacunosum-moleculare	463	495	CA3sp	Field CA3, pyramidal layer	463
872	DR	Dorsal nucleus raphe	165	100	IPN	Interpeduncular nucleus	165
460	MEV	Midbrain trigeminal nucleus	339	580	NB	Nucleus of the brachium of the inferior colliculus	339
162	LDT	Laterodorsal tegmental nucleus	1117	358	SLD	Sublaterodorsal nucleus	1117
757	VTN	Ventral tegmental nucleus	323	246	RR	Midbrain reticular nucleus, retrorubral area	323
523	MPO	Medial preoptic area	141	347	SBPV	Subparaventricular zone	141
523	MPO	Medial preoptic area	141	126	PVp	Periventricular hypothalamic nucleus, posterior part	141
1010	VISC4	Visceral area, layer 4	677	1058	VISC5	Visceral area, layer 5	677
1105	IA	Intercalated amygdalar nucleus	278	23	AAA	Anterior amygdalar area	278
149	PVT	Paraventricular nucleus of the thalamus	571	15	PT	Parataenial nucleus	571
604	NI	Nucleus incertus	1117	238	RPO	Nucleus raphe pontis	1117
564	MS	Medial septal nucleus	904	596	NDB	Diagonal band nucleus	904
860	PBlc	Parabrachial nucleus, lateral division, central lateral part	881	875	PBle	Parabrachial nucleus, lateral division, external lateral part	881
860	PBlc	Parabrachial nucleus, lateral division, central lateral part	881	891	PBlv	Parabrachial nucleus, lateral division, ventral lateral part	881
800	AIv5	Agranular insular area, ventral part, layer 5	119	704	AIv1	Agranular insular area, ventral part, layer 1	119
907	PCN	Paracentral nucleus	51	599	CM	Central medial nucleus of the thalamus	51
335	PERI6a	Perirhinal area, layer 6a	922	888	PERI2/3	Perirhinal area, layer 2/3	922
84	PL6a	Prelimbic area, layer 6a	972	363	PL5	Prelimbic area, layer 5	972
724	AHNp	Anterior hypothalamic nucleus, posterior part	88	708	AHNc	Anterior hypothalamic nucleus, central part	88
347	SBPV	Subparaventricular zone	141	126	PVp	Periventricular hypothalamic nucleus, posterior part	141
591	CLI	Central linear nucleus raphe	165	100	IPN	Interpeduncular nucleus	165
973	VISl2/3	Lateral visual area, layer 2/3	409	421	VISl1	Lateral visual area, layer 1	409
810	ACAv6a	Anterior cingulate area, ventral part, 6a	48	588	ACAv1	Anterior cingulate area, ventral part, layer 1	48
700	AHNa	Anterior hypothalamic nucleus, anterior part	88	708	AHNc	Anterior hypothalamic nucleus, central part	88
232	COApl3	Cortical amygdalar area, posterior part, lateral zone, layer 3	655	224	COApl2	Cortical amygdalar area, posterior part, lateral zone, layer 2	655
377	VISpl6a	Posterolateral visual area, layer 6a	425	902	VISpl5	Posterolateral visual area, layer 5	425
1125	ORBvl5	Orbital area, ventrolateral part, layer 5	746	288	ORBvl2/3	Orbital area, ventrolateral part, layer 2/3	746
845	SUBd-sp	Subiculum, dorsal part, pyramidal layer	509	829	SUBd-m	Subiculum, dorsal part, molecular layer	509
1015	ACAd5	Anterior cingulate area, dorsal part, layer 5	39	919	ACAd6a	Anterior cingulate area, dorsal part, layer 6a	39
216	COApl1	Cortical amygdalar area, posterior part, lateral zone, layer 1	655	224	COApl2	Cortical amygdalar area, posterior part, lateral zone, layer 2	655
613	VISl5	Lateral visual area, layer 5	409	421	VISl1	Lateral visual area, layer 1	409
10693	PAR1	Parasubiculum, layer 1	843	10695	PAR3	Parasubiculum, layer 3	843
56	ACB	Nucleus accumbens	493	998	FS	Fundus of striatum	493
10701	PRE3	Presubiculum, layer 3	1084	10700	PRE2	Presubiculum, layer 2	1084
664	ENTm3	Entorhinal area, medial part, dorsal zone, layer 3	926	727	ENTm5	Entorhinal area, medial part, dorsal zone, layer 5	926
358	SLD	Sublaterodorsal nucleus	1117	238	RPO	Nucleus raphe pontis	1117
268	NLOT2	Nucleus of the lateral olfactory tract, pyramidal layer	619	1139	NLOT3	Nucleus of the lateral olfactory tract, layer 3	619
268	NLOT2	Nucleus of the lateral olfactory tract, pyramidal layer	619	260	NLOT1	Nucleus of the lateral olfactory tract, molecular layer	619
479	CA3slu	Field CA3, stratum lucidum	463	495	CA3sp	Field CA3, pyramidal layer	463
511	SCig-c	Superior colliculus, motor related, intermediate gray layer, su	10	494	SCig-a	Superior colliculus, motor related, intermediate gray layer, sul	10
310	SF	Septofimbrial nucleus	275	333	SH	Septohippocampal nucleus	275
484	ORBm1	Orbital area, medial part, layer 1	731	620	ORBm5	Orbital area, medial part, layer 5	731
638	GU6a	Gustatory areas, layer 6a	1057	662	GU6b	Gustatory areas, layer 6b	1057
15	PT	Parataenial nucleus	571	181	RE	Nucleus of reunions	571
478	SSp-ll6a	Primary somatosensory area, lower limb, layer 6a	337	510	SSp-ll6b	Primary somatosensory area, lower limb, layer 6b	337
598	AUDv6b	Ventral auditory area, layer 6b	1018	1023	AUDv5	Ventral auditory area, layer 5	1018

137	CSl	Superior central nucleus raphe, lateral part	679	130	CSm	Superior central nucleus raphe, medial part	679
727	ENTm5	Entorhinal area, medial part, dorsal zone, layer 5	926	743	ENTm6	Entorhinal area, medial part, dorsal zone, layer 6	926
450	SSp-ul1	Primary somatosensory area, upper limb, layer 1	369	854	SSp-ul2/3	Primary somatosensory area, upper limb, layer 2/3	369
1139	NLOT3	Nucleus of the lateral olfactory tract, layer 3	619	260	NLOT1	Nucleus of the lateral olfactory tract, molecular layer	619
440	ORBl6a	Orbital area, lateral part, layer 6a	723	630	ORBl5	Orbital area, lateral part, layer 5	723
