# Supplementary material for: Assessing the replicability of spatial gene expression using atlas data from the adult mouse brain
Source: PLoS Biol. 2021 Jul 19;19(7):e3001341. doi: 10.1371/journal.pbio.3001341 (PMC8321401; doi:10.1371/journal.pbio.3001341)
Supplement: S3 Table — ABA, Allen Brain Atlas; AUROC, area under the receiver operating curve; LASSO, least absolute shrinkage and selection operator; ST, spatial transcriptomics. (PDF) [file pbio.3001341.s014.pdf]

Supplementary Table 3

ABA to ST LASSO, alpha=0.1 auroc file = "ABAtoST_STall_f1_0p1_051420.csv"

AUROC >= 0.95; path length = 2

Brain Area 1				Brain Area 2			
id	acronym	name	parent	id	acronym	name	parent
1005	AUDp6b	Primary auditory area, layer 6b	1002	816	AUDp4	Primary auditory area, layer 4	1002
943	MOp2/3	Primary motor area, Layer 2/3	985	882	MOp6b	Primary motor area, Layer 6b	985
882	MOp6b	Primary motor area, Layer 6b	985	320	MOp1	Primary motor area, Layer 1	985
269	VISpl2/3	Posterolateral visual area, layer 2/3	425	377	VISpl6a	Posterolateral visual area, layer 6a	425
1045	ECT6b	Ectorhinal area/Layer 6b	895	836	ECT1	Ectorhinal area/Layer 1	895

AUROC <=0.5; path length = 2

Brain Area 1				Brain Area 2			
id	acronym	name	parent	id	acronym	name	parent
657	SSp-m2/3	Primary somatosensory area, mouth, layer 2/3	345	950	SSp-m4	Primary somatosensory area, mouth, layer 4	345
1114	VISal4	Anterolateral visual area, layer 4	402	233	VISal5	Anterolateral visual area, layer 5	402
606	RSPv2	Retrosplenial area, ventral part, layer 2	886	622	RSPv6b	Retrosplenial area, ventral part, layer 6b	886
606	RSPv2	Retrosplenial area, ventral part, layer 2	886	430	RSPv2/3	Retrosplenial area, ventral part, layer 2/3	886
472	MEApd-a	Medial amygdalar nucleus, posterodorsal part, sublayer a	426	487	MEApd-c	Medial amygdalar nucleus, posterodorsal part, sublayer c	426
472	MEApd-a	Medial amygdalar nucleus, posterodorsal part, sublayer a	426	480	MEApd-b	Medial amygdalar nucleus, posterodorsal part, sublayer b	426
980	PMd	Dorsal premammillary nucleus	467	946	PH	Posterior hypothalamic nucleus	467
980	PMd	Dorsal premammillary nucleus	467	1004	PMv	Ventral premammillary nucleus	467
296	ACAv2/3	Anterior cingulate area, ventral part, layer 2/3	48	772	ACAv5	Anterior cingulate area, ventral part, layer 5	48
148	GU4	Gustatory areas, layer 4	1057	187	GU5	Gustatory areas, layer 5	1057
148	GU4	Gustatory areas, layer 4	1057	662	GU6b	Gustatory areas, layer 6b	1057
783	AId6a	Agranular insular area, dorsal part, layer 6a	104	1101	AId5	Agranular insular area, dorsal part, layer 5	104
381	SNr	Substantia nigra, reticular part	323	616	CUN	Cuneiform nucleus	323
381	SNr	Substantia nigra, reticular part	323	757	VTN	Ventral tegmental nucleus	323
191	AONm	Anterior olfactory nucleus, medial part	159	167	AONd	Anterior olfactory nucleus, dorsal part	159
416	PAA2	Piriform-amygdalar area, pyramidal layer	788	424	PAA3	Piriform-amygdalar area, polymorph layer	788
868	PBld	Parabrachial nucleus, lateral division, dorsal lateral part	881	860	PBlc	Parabrachial nucleus, lateral division, central lateral part	881
868	PBld	Parabrachial nucleus, lateral division, dorsal lateral part	881	891	PBlv	Parabrachial nucleus, lateral division, ventral lateral part	881
1106	VISC2/3	Visceral area, layer 2/3	677	1058	VISC5	Visceral area, layer 5	677
628	NOT	Nucleus of the optic tract	1100	634	NPC	Nucleus of the posterior commissure	1100
628	NOT	Nucleus of the optic tract	1100	215	APN	Anterior pretectal nucleus	1100
105	SOCm	Superior olivary complex, medial part	398	122	POR	Superior olivary complex, periolivary region	398
194	LHA	Lateral hypothalamic area	290	364	PSTN	Parasubthalamic nucleus	290
1062	SSp-bfd6b	Primary somatosensory area, barrel field, layer 6b	329	1070	SSp-bfd5	Primary somatosensory area, barrel field, layer 5	329
465	OT2	Olfactory tubercle, pyramidal layer	754	473	OT3	Olfactory tubercle, polymorph layer	754
465	OT2	Olfactory tubercle, pyramidal layer	754	481	isl	Islands of Calleja	754
1102	SSp-m6a	Primary somatosensory area, mouth, layer 6a	345	878	SSp-m1	Primary somatosensory area, mouth, layer 1	345
1102	SSp-m6a	Primary somatosensory area, mouth, layer 6a	345	2	SSp-m6b	Primary somatosensory area, mouth, layer 6b	345
189	RH	Rhomboid nucleus	51	575	CL	Central lateral nucleus of the thalamus	51
694	AIv2/3	Agranular insular area, ventral part, layer 2/3	119	800	AIv5	Agranular insular area, ventral part, layer 5	119
344	AIp5	Agranular insular area, posterior part, layer 5	111	314	AIp6a	Agranular insular area, posterior part, layer 6a	111
965	RSPagl2/3	Retrosplenial area, lateral agranular part, layer 2/3	894	774	RSPagl5	Retrosplenial area, lateral agranular part, layer 5	894
272	AVPV	Anteroventral periventricular nucleus	141	523	MPO	Medial preoptic area	141
272	AVPV	Anteroventral periventricular nucleus	141	347	SBPV	Subparaventricular zone	141
263	AVP	Anteroventral preoptic nucleus	141	286	SCH	Suprachiasmatic nucleus	141
263	AVP	Anteroventral preoptic nucleus	141	523	MPO	Medial preoptic area	141
263	AVP	Anteroventral preoptic nucleus	141	126	PVp	Periventricular hypothalamic nucleus, posterior part	141
458	OT1	Olfactory tubercle, molecular layer	754	481	isl	Islands of Calleja	754
687	RSPv5	Retrosplenial area, ventral part, layer 5	886	430	RSPv2/3	Retrosplenial area, ventral part, layer 2/3	886
292	BA	Bed nucleus of the accessory olfactory tract	278	1105	IA	Intercalated amygdalar nucleus	278
635	PTLp4	Posterior parietal association areas, layer 4	22	241	PTLp2/3	Posterior parietal association areas, layer 2/3	22
683	PTLp5	Posterior parietal association areas, layer 5	22	241	PTLp2/3	Posterior parietal association areas, layer 2/3	22
622	RSPv6b	Retrosplenial area, ventral part, layer 6b	886	590	RSPv6a	Retrosplenial area, ventral part, layer 6a	886
622	RSPv6b	Retrosplenial area, ventral part, layer 6b	886	430	RSPv2/3	Retrosplenial area, ventral part, layer 2/3	886
1086	SSp-tr4	Primary somatosensory area, trunk, layer 4	361	461	SSp-tr6b	Primary somatosensory area, trunk, layer 6b	361
305	VISp6b	Primary visual area, layer 6b	385	33	VISp6a	Primary visual area, layer 6a	385
837	SUBd-sr	Subiculum, dorsal part, stratum radiatum	509	845	SUBd-sp	Subiculum, dorsal part, pyramidal layer	509
544	CEAc	Central amygdalar nucleus, capsular part	536	551	CEAl	Central amygdalar nucleus, lateral part	536
411	MEAad	Medial amygdalar nucleus, anterodorsal part	403	418	MEAav	Medial amygdalar nucleus, anteroventral part	403
614	TU	Tuberal nucleus	290	173	RCH	Retrochiasmatic area	290
614	TU	Tuberal nucleus	290	226	LPO	Lateral preoptic area	290
187	GU5	Gustatory areas, layer 5	1057	662	GU6b	Gustatory areas, layer 6b	1057
41	VISpm2/3	posteromedial visual area, layer 2/3	533	565	VISpm5	posteromedial visual area, layer 5	533
303	BLAa	Basolateral amygdalar nucleus, anterior part	295	451	BLAv	Basolateral amygdalar nucleus, ventral part	295
654	SSp-n4	Primary somatosensory area, nose, layer 4	353	838	SSp-n2/3	Primary somatosensory area, nose, layer 2/3	353
266	LSv	Lateral septal nucleus, ventral part	242	258	LSr	Lateral septal nucleus, rostral (rostroventral) part	242
304	PL2/3	Prelimbic area, layer 2/3	972	363	PL5	Prelimbic area, layer 5	972
646	DP5	Dorsal peduncular area, layer 5	814	360	DP2/3	Dorsal peduncular area, layer 2/3	814
412	ORBl2/3	Orbital area, lateral part, layer 2/3	723	448	ORBl1	Orbital area, lateral part, layer 1	723
616	CUN	Cuneiform nucleus	323	757	VTN	Ventral tegmental nucleus	323
772	ACAv5	Anterior cingulate area, ventral part, layer 5	48	588	ACAv1	Anterior cingulate area, ventral part, layer 1	48
286	SCH	Suprachiasmatic nucleus	141	347	SBPV	Subparaventricular zone	141
486	CA3so	Field CA3, stratum oriens	463	471	CA3slm	Field CA3, stratum lacunosum-moleculare	463
1075	TTv2	Taenia tecta, ventral part, layer 2	605	1082	TTv3	Taenia tecta, ventral part, layer 3	605
692	PERI5	Perirhinal area, layer 5	922	335	PERI6a	Perirhinal area, layer 6a	922
501	VISpm4	posteromedial visual area, layer 4	533	565	VISpm5	posteromedial visual area, layer 5	533
233	VISal5	Anterolateral visual area, layer 5	402	649	VISal6b	Anterolateral visual area, layer 6b	402
233	VISal5	Anterolateral visual area, layer 5	402	601	VISal6a	Anterolateral visual area, layer 6a	402
520	AUDv6a	Ventral auditory area, layer 6a	1018	598	AUDv6b	Ventral auditory area, layer 6b	1018
1045	ECT6b	Ectorhinal area/Layer 6b	895	977	ECT6a	Ectorhinal area/Layer 6a	895
52	ENTl3	Entorhinal area, lateral part, layer 3	918	715	ENTl2a	Entorhinal area, lateral part, layer 2a	918

473	OT3	Olfactory tubercle, polymorph layer	754	481	isl	Islands of Calleja	754
712	ENTm4	Entorhinal area, medial part, dorsal zone, layer 4	926	664	ENTm3	Entorhinal area, medial part, dorsal zone, layer 3	926
883	PBls	Parabrachial nucleus, lateral division, superior lateral part	881	891	PBlv	Parabrachial nucleus, lateral division, ventral lateral part	881
471	CA3slm	Field CA3, stratum lacunosum-moleculare	463	495	CA3sp	Field CA3, pyramidal layer	463
670	SSp-tr2/3	Primary somatosensory area, trunk, layer 2/3	361	461	SSp-tr6b	Primary somatosensory area, trunk, layer 6b	361
872	DR	Dorsal nucleus raphe	165	591	CLI	Central linear nucleus raphe	165
460	MEV	Midbrain trigeminal nucleus	339	580	NB	Nucleus of the brachium of the inferior colliculus	339
537	BSTal	Bed nuclei of the stria terminalis, anterior division, anterolateral area	359	498	BSTam	Bed nuclei of the stria terminalis, anterior division, anteromedial area	359
1094	SSp-ll4	Primary somatosensory area, lower limb, layer 4	337	510	SSp-ll6b	Primary somatosensory area, lower limb, layer 6b	337
162	LDT	Laterodorsal tegmental nucleus	1117	358	SLD	Sublaterodorsal nucleus	1117
162	LDT	Laterodorsal tegmental nucleus	1117	238	RPO	Nucleus raphe pontis	1117
757	VTN	Ventral tegmental nucleus	323	749	VTA	Ventral tegmental area	323
757	VTN	Ventral tegmental nucleus	323	246	RR	Midbrain reticular nucleus, retrorubral area	323
757	VTN	Ventral tegmental nucleus	323	214	RN	Red nucleus	323
889	SSp-n6a	Primary somatosensory area, nose, layer 6a	353	702	SSp-n5	Primary somatosensory area, nose, layer 5	353
149	PVT	Paraventricular nucleus of the thalamus	571	15	PT	Parataenial nucleus	571
604	NI	Nucleus incertus	1117	358	SLD	Sublaterodorsal nucleus	1117
307	MARN	Magnocellular reticular nucleus	370	661	VII	Facial motor nucleus	370
907	PCN	Paracentral nucleus	51	599	CM	Central medial nucleus of the thalamus	51
649	VISal6b	Anterolateral visual area, layer 6b	402	601	VISal6a	Anterolateral visual area, layer 6a	402
724	AHNp	Anterior hypothalamic nucleus, posterior part	88	708	AHNc	Anterior hypothalamic nucleus, central part	88
591	CLI	Central linear nucleus raphe	165	100	IPN	Interpeduncular nucleus	165
487	MEApd-c	Medial amygdalar nucleus, posterodorsal part, sublayer c	426	480	MEApd-b	Medial amygdalar nucleus, posterodorsal part, sublayer b	426
232	COApl3	Cortical amygdalar area, posterior part, lateral zone, layer 3	655	224	COApl2	Cortical amygdalar area, posterior part, lateral zone, layer 2	655
377	VISpl6a	Posterolateral visual area, layer 6a	425	902	VISpl5	Posterolateral visual area, layer 5	425
454	CA2sr	Field CA2, stratum radiatum	423	446	CA2sp	Field CA2, pyramidal layer	423
1113	IAD	Interanterodorsal nucleus of the thalamus	239	155	LD	Lateral dorsal nucleus of thalamus	239
503	SCig-b	Superior colliculus, motor related, intermediate gray layer, sublayer b	10	511	SCig-c	Superior colliculus, motor related, intermediate gray layer, sublayer c	10
634	NPC	Nucleus of the posterior commissure	1100	215	APN	Anterior pretectal nucleus	1100
613	VISl5	Lateral visual area, layer 5	409	421	VISl1	Lateral visual area, layer 1	409
56	ACB	Nucleus accumbens	493	998	FS	Fundus of striatum	493
578	BSTpr	Bed nuclei of the stria terminalis, posterior division, principal nucleus	367	585	BSTif	Bed nuclei of the stria terminalis, posterior division, interfascicular nucleus	367
676	DMHp	Dorsomedial nucleus of the hypothalamus, posterior part	830	668	DMHa	Dorsomedial nucleus of the hypothalamus, anterior part	830
360	DP2/3	Dorsal peduncular area, layer 2/3	814	496	DP1	Dorsal peduncular area, layer 1	814
479	CA3slu	Field CA3, stratum lucidum	463	495	CA3sp	Field CA3, pyramidal layer	463
511	SCig-c	Superior colliculus, motor related, intermediate gray layer, sublayer c	10	494	SCig-a	Superior colliculus, motor related, intermediate gray layer, sublayer a	10
778	VISp5	Primary visual area, layer 5	385	721	VISp4	Primary visual area, layer 4	385
310	SF	Septofimbrial nucleus	275	333	SH	Septohippocampal nucleus	275
638	GU6a	Gustatory areas, layer 6a	1057	662	GU6b	Gustatory areas, layer 6b	1057
764	ENTl2b	Entorhinal area, lateral part, layer 2b	918	715	ENTl2a	Entorhinal area, lateral part, layer 2a	918
1046	VISam6a	Anteromedial visual area, layer 6a	394	441	VISam6b	Anteromedial visual area, layer 6b	394
668	DMHa	Dorsomedial nucleus of the hypothalamus, anterior part	830	684	DMHv	Dorsomedial nucleus of the hypothalamus, ventral part	830
1127	TEa2/3	Temporal association areas, layer 2/3	541	234	TEa4	Temporal association areas, layer 4	541
875	PBle	Parabrachial nucleus, lateral division, external lateral part	881	891	PBlv	Parabrachial nucleus, lateral division, ventral lateral part	881
1096	AMd	Anteromedial nucleus, dorsal part	127	1104	AMv	Anteromedial nucleus, ventral part	127
440	ORBl6a	Orbital area, lateral part, layer 6a	723	630	ORBl5	Orbital area, lateral part, layer 5	723
