# Supplementary material for: Assessing the replicability of spatial gene expression using atlas data from the adult mouse brain
Source: PLoS Biol. 2021 Jul 19;19(7):e3001341. doi: 10.1371/journal.pbio.3001341 (PMC8321401; doi:10.1371/journal.pbio.3001341)
Supplement: Attachment — Submitted filename: PLOS_ResponsetoReviewers_052921.docx [file pbio.3001341.s015.docx]

We’re grateful for two highly thoughtful reviews from the two ends of the spectrum of our intended audience (bioinformatics and neuroscience). We were pleased to see that both reviewers had highly positive impressions of the manuscript and underlying work. In response to their comments and queries, we have performed new experiments (generally serving to highlight the robustness of our results) and made significant additions to improve the narrative. In the following, we leave comments from reviewers in regular “black” with our responses in blue. (For papers referenced here, full citations are available in the main text.)

Unfortunately, due to a Microsoft Word bug, when the main text is converted to a PDF (for both main text and track changes version), line numbers greater than 635 jump up by 21 lines (transition between pages 28-29). Here we indicate these line numbers with the jump as well as the original in parentheticals.

REVIEWS:

Reviewer #1: This is a well-written paper on a timely topic. Spatial gene expression analyses are currently becoming increasingly important, and the question of their replicability/generalizability is a crucial one.

However, I have a couple of minor and major concerns:

1. It would be good to clearly define the term "benchmarking".

We agree that we use the term “benchmarking” loosely in our manuscript. We have updated the manuscript to include a brief definition in the introduction on lines 117-118 (page 5). We appreciate that benchmarking in computational biology generally refers to the comparison of algorithms/software. However, we feel that since neuroscience is unusual in comparison to many other fields in having large central resources (ABA) akin to the reference genome in genomics, that there was a need to “benchmark” the data itself. So, here we instead hold the analyses tools constant to compare the data itself as representatives of different experimental assays.

2. The role of batch effects could be discussed in more details, both within datasets and across datasets. In other settings, it has been shown that correction for batch effects may improve cross-study classification. Could it also be the case here?

This is an interesting question that we were happy to investigate more fully in the cross dataset case. In our case, detecting batches within datasets is not conventionally possible. Particularly, in the ABA, each gene is measured in a separate experiment, so the batches are the genes which are also the features themselves. Since the ABA is collected using single molecule *in situ hybridization*, each gene must be collected as a separate experiment.

We did, however, follow-up on the suggestion of further exploring the role of batch correction in the cross-dataset case. Treating each dataset as a batch, we applied a python implementation of ComBat to a combined matrix (Behdenna *et al.*, 2020 bioRxiv). We observed little to no effect of batch correction on cross-dataset performance which was further backed by the lack of visible batches when the two datasets were visualized in low-dimensional space (Supplementary Figure 6a-c). We included these results on lines 312-330 (pages 14-15) in the results and 743-748 (722-727) (page 33) in the methods.

In addition, it is worth noting that the main use case for these atlases is as a reference for more targeted experiments (i.e. for comparison of *in situ hybridization* images), so it is often not possible to globally integrate new data (which may consist of a single gene). We are using two global datasets to assess consistency for the harder case where batch correction is not straightforwardly possible.

3. p5: It would be helpful to summarize the format of the data (how many rows, columns, and what they correspond to).
Good suggestion, we added a sentence on lines 167-170 (page 7) to the first paragraph of the results briefly detailing the data and referencing the methods for more information on preliminarily and subsequent filtering. For reference: “the ABA consists of 62,527 voxels (rows) with expression from 19,934 unique genes (columns) mapping to 569 non-overlapping brain area labels and the ST consists of 30,780 spots (rows) with 16,557 genes (columns) mapping to 461 brain area labels (see methods for details).”

4. As far as I can understand, each dataset corresponds to one brain. It would be good to clearly state that, or to clarify the text if I am wrong.
No, we apologize for not stating this information clearly in the text. Based on our estimation from the methods of Lein *et al.,* 2007, the ABA consists of a minimum of 3,260 brains, while the ST dataset consists of 3 brains (Ortiz *et al.*, 2020). We have updated this on lines 167-170 (page 7) in the results for both datasets and on lines 593-595 (page 27) in the methods for the ABA. The number of brains in the ST dataset was also previously indicated in the methods on line 580 (page 26).

5. Did the authors consider using dedicated multi-class classification methods rather than considering pairwise comparisons?
Preferring the ease of interpretability of multiple pairwise classifications, we had not originally considered dedicated multi-class classification methods. However, we were very happy to explore this suggestion.

Building from our approach, we first tried to do a one v. rest (OVR) classification using linear regression, since LASSO fails for many one v. rest comparisons (Supplementary Figure 5). To turn this into an OVR multiclass classification we took the predictions vectors, re-ranked across the samples (rows) then re-calculate an AUROC from each column. We tried both ranking and not ranking the initial prediction vectors prior to sample ranking which made no discernable difference, so we report the ranked results here. Performance falls relative to the non-multiclass linear regression mean for ABA (non-multiclass mean AUROC = 0.951 to OVR multiclass mean AUROC = 0.579), but is very similar in ST (non-multiclass mean AUROC = 0.613 to OVR multiclass mean AUROC = 0.615).

In addition to OVR, we also explored using the k-nearest neighbors (k-NN) algorithm with default parameters (k=5). We again found that classification performance drops for both datasets (ABA mean AUROC = 0.695; ST mean AUROC = 0.508). This is described in the manuscript on lines 211-218 (page 9) and in the methods on lines 729-741 (708-720) (page 32). We acknowledge there is absolutely room for other algorithmic approaches to be developed that might be an improvement over these somewhat default approaches; we hope that we have set a reasonable baseline that establishes improvement would be an important contribution.

6. The regularization parameter in lasso is usually called lambda.
Thank you for pointing this out; we accordingly replaced “alpha” with “lambda” in all use cases.

7. Two PCs capturing as much as 80% of the variance seems suspicious to me. Is it possible that large batch effects are an issue for this dataset, i.e. that data different brain regions have been collected/preprocessed in different ways, which would explain the very good classification accuracy?

We apologize, as we were unclear in the manuscript that the result we were reporting was for each brain area individually. In other words, we perform PCA in each brain area separately and find that across all brain areas, in ABA an average of 2 PCs captured 80% variance for its respective brain area. We clarified the manuscript text accordingly on line 255-259 (page 11). In this scenario, this result does not indicate batch effects because they are not the same two PCs across brain areas. It does suggest that some post-hoc registration within each brain area may be overfitting (the hypothesis we discuss with respect to the ABA data).

As to your point, we completely agree that two PC’s capturing 80% of the variance across the whole dataset would indicate large batch effects. Applying PCA to the whole dataset, we see that this is not the case (Supplementary Figure 3e). We added this result on lines 260-262 (page 11) of the results.

Finally, responding to your example, we also completely agree that if different brain regions were collected/processed in different ways that we would observe the classification accuracy that we do. However, while brains are processed in slices for both ST and ABA datasets (the predominate approach in neuroscience), different areas, with the exception of the fact that not all brain areas are represented in each slice, do not represent different batches. Most brain areas span multiple slices. If anything, the genes would be more likely to represent batches in the ABA where each gene is collected in a separate single molecule in situ hybridization experiment in different tissue (interlayered slices to collect ~8 genes per brain across many brains for the full dataset). At the risk of redundancy in our explanation: each gene in the ABA is collected in different physical samples.

8. p11: "we picked 100 randomly seeded feature sets": How large are these feature sets?
We apologize for omitting this information originally. We added text to the methods on lines 773-775 (752-754) (page 34) with the range (ABA range 1-29; ST range 1-47) and median feature set sizes (ABA median size = 2; ST median size = 4) and added supplementary figure 7a,b to show the distribution of feature set sizes.

9. p16: I don’t understand how the authors can apply simple linear regression to this dataset. Are the data not high-dimensional?

The data are indeed high dimensional. In this paradigm, where there are more features than samples, there are many possible solutions to linear regression so it is underdetermined as the reviewer pointed out. We use the scikit-learn implementation which, instead of simply returning linear regression as not uniquely unsolvable, selects the solution with the minimum Euclidean norm (of its coefficients). (This is different from Ridge Regression where the L2 norm is incorporated in the cost function.) We have clarified this in the methods on lines 722-727 (701-706) (page 32).

10. p22: The authors used 2-fold CV. That is fine, but I am wondering whether the results are stable. Would they have been the same with another random split into training and test data?
Yes, the results are stable across a second random train/test split. For all of our results originally presented in the manuscript, we seeded our random splits with the integer 42 (all random functions in python are actually pseudorandom). To demonstrate the stability, we re-did both the within dataset analysis for ST and ABA as documented on lines 203-205 (page 9) (Supplementary Figure 1c-f) and the cross dataset analysis on lines 303-308 (pages 13-14) (Supplementary Figure 5a-c) with a seed of 9. The results with the new seed have very similar mean AUROCs with the original seed in all cases.

11. p22: "For within ST training and testing,...": It is a bit confusing to repeat that, since it has already been said at the beginning of the paragraph (reading this I got confused and wondered whether the sentence before was only about ST or general).
Sorry about the confusion, we can completely see why that lead-in can be misleading and removed it accordingly.

12. The cross-dataset analysis is based on the 14,299 overlapping genes, i.e. on a subset of genes. It would be interesting to see the within dataset performance obtained using only these genes, to check that the worse performance in cross-dataset learning is not due to important genes being absent.
We are sorry for the confusion in our explanation as we completely agree and this is indeed what we do. The comparisons in figure 3 of the cross-dataset performance (blue) and within dataset test performance (purple) are a matched comparison- they both only use the subset of genes. We also added an additional sentence to the methods to clarify this on lines 296-298 (page 13) and in the results on lines 632-634 (page 28).

13. p23: "The AUROC is typically...": This sentence is already about the *area under* the ROC curve, so the following sentence is redundant. Note that the "analytical AUROC" (as the authors call it) is also an estimate of the true unknown AUROC.
Great catch, thank you, we removed the second sentence accordingly.

Thank you also for your note as it helped to clean up the communication of our methods. When we say the AUROC calculated as we do is accurate, we are referring to the calculation on the sample that we see. We have clarified this in the sentence on line 663-664 (642-643) (page 29). We agree that it is still an estimate of the true unknown population AUROC.

14. p23: The AUROC formula given by the authors is not a standard one. Please give a standard one or specify a reference.
The AUROC formula that we give is based on the relationship between the Mann-Whitney U-statistic and the AUROC. This link is well established in the literature (Hanley and McNeil 1982; Mason and Graham 2002; Krzanowski and Hand 2009).

If we define the AUROC based on the U-statistic:

$$AUROC= \frac{U}{N_{Pos}N_{Neg}}$$

And the U-statistic as:

$$U=\sum_{i}^{N} {Ranks}_{i}- \frac{N_{Pos}(N_{Pos}+1)}{2}$$

Substituting the equation for U in the first AUROC formula we get the equation presented in our methods section:
15. p23: Why were AUROCs of 0 removed from downstream reporting? This may introduce a bias. What about values between 0 and 0.5?
We are truly indebted to the reviewer for pointing this out. This was extremely sloppy on our part and a mistake held over from an early draft. We have updated the methods on lines 671-673 (650-652) (page 29) to fix this. In most cases (cross-dataset LASSO, cross-dataset CFS, cross planes, and one to all analysis (figure 5)), we do not remove the AUROCs of 0 from downstream reporting.

$$AUROC= \sum_{i}^{N} \frac{{Ranks}_{i}}{N_{Pos} *N_{Neg}} - \frac{N_{Pos}+1}{2 * N_{Neg}}$$

The only case that we actually filter out zeros was in the visualization and reporting of means for the within dataset AUROC for ST and ABA (Figure 2). In this case there are very few AUROCs of 0 (i.e. 22 pairs for ST and 4 pairs for ABA when lambda = 0.1) and those that do occur only occur in very small brain areas. (As an example, for lambda = 0.1, the max combined sample size for a brain area pair giving an AUROC = 0 in ST was 19 and in ABA was 17.) This extremely small sample size combined with low dimensionality can result in highly unusual AUROCs. In fact, since we have 2 folds, we actually observe that the AUROCs of 0 do not occur for the same brain area pairs across folds for all pairs in the ST and for all but one pair in the ABA when lambda = 0.1. Most importantly, removing or leaving the AUROCs of 0 do not affect the means to the third decimal place, which is what is reported in the manuscript.

For Figure 2c, we also checked the AUROCs of 0 across the other lambdas. We observe a similar phenomenon for LASSO with lambda = 0.01, lambda = 0.05, and linear regression. Again, removing or leaving the AUROCs of 0 do not affect the means to the third decimal place for all parameterizations, which is what is reported in the manuscript.

AUROCs between 0 and 0.5 are not filtered out and are included in all distributions and downstream reporting.

16. p23: "We use a threshold that includes all instances of the positive label". I don’t understand that. How do the authors choose with class of the pair is the "positive one"? Or do they perform two analyses successively for each pair?
Sorry that this was unclear. We changed the text on line 680 (659) (page 30) to “one class” instead of “the positive label.” We simply refer to one class as the positive class. It does not matter which class is referred to as the positive class as long we are consistent. We simply chose this language to map to the true positive/true negative/false positive/false negative language.

17. p23: "Note, that for while": typo
Thank you! (Fixed on line 683 (662) (page 30).)

18. p23: What is the rationale behind the statement at the end of p23/beginning of p24?
Apologies about the confusion; this was purely trying to clarify the coding. To calculate the median performance, we use the averaged AUROC across the two folds. However, this gives a median value that does not exactly map to a pair of brain areas in either fold. So, to calculate the median precision, we find a pair(s) of brain areas that have the median performance in that fold and then average this precision across all pairs between the two folds. We modified the methods on lines 683-686 (662-665) (page 30) to clear this up.

19. p24: It is bad practice to set the penalty parameter to an arbitrary value. This parameter has a huge importance on the performance of the model. It should be chosen using cross-validation. Otherwise, one cannot guarantee that, for example, the better accuracy for ABA is due to 0.1 being more appropriate for this dataset than for ST.
We completely agree that it is bad practice to pick an arbitrary penalty hyperparameter. Though not formally, we did try a variety of hyperparameters showing the results of cross-validation in these figures: Figure 2c, Figure 5b, Supplementary Figure 10. The original statement in the methods was a poor choice of language on our part; we simply meant to express that the actual hyperparameter value used is as indicated in the main text.

Regardless, we recognize that our hyperparameter selection was not properly formalized and went back to do this. We have expanded on our LASSO methods section on lines 699-720 (678-699) (page 31) with accompanying figures (Supplementary Figure 11). In summary, we did hyperparameter selection on a subset of area with sufficient sample size and then chose a global hyperparameter for our analysis based on this. Confirming our approach, we compared the three-way cross-dataset analysis on a subset of brain areas using (1) a dynamic hyperparameter selected for each brain area pair classification to (2) using a fixed lambda. We observed that performance was comparable in either case (Supplementary Figure 8c-d). In summary for our case, conclusions drawn, regardless of hyperparameter choice, remain the same.

20. p24: Please define the notation ||.||1.
Done! (on line 694-695 (673-674) (page 30))

21. p24: "PCA was applied [...] to each leaf brain". I don’t understand that. Did the authors really repeat the PCA analyses for each leaf brain successively? Does that make sense? If yes, how did
they compute the reported proportion of explained variability?
Yes, that is exactly what we did. We performed PCA for each brain area separately. We counted the minimum number of PCs needed to capture at least 80% of the variability in each brain area. The distribution of these counts across brain areas is shown in Figure 2D and the reported number of PCs is the mean of this distribution.

Additionally, Supplementary Figure 3d attempts to show the cumulative explained variance as a function of dimensions for each of the leaf brain areas across the two datasets. There are many curves shown, but the viewer gets an idea of the general trends.

We performed this experiment in this manner as a targeted experiment, since we suspected that one of the causes for high classification performance in the ABA could be due to overfitting during iterative registration of the samples to the reference brain. Global PCs might not capture this, so we split it out into individual brain areas. However, for completion, we also added a results on applying PCA (Supplementary Figure 3e) to the whole brain detailed in response to point 7 above and Reviewer 2 below.

Reviewer #2: This work compares the identifiability of anatomic structures by gene expression comparing in situ hybridization data from the Allen Mouse Brain Atlas to modern spatial transcriptomic data capable of labeling cells expressing specific genes. The study is in principle well-conceived in that it very useful to compare cell type specific expression patterns and their anatomic identifiability between these modalities. The potential for one to inform the other is both important and interesting. This study in this way is very timely in comparing a widely used standard data set with more modern powerful expression profiling to understand key cellular co-expression relationships. The success of this method, in particular using the expression energy of the 200 cubic micron grid data is both interesting and surprising. In a sense this grid data is highly smoothed and a very coarse representation of the cell specific expression in the ABA. Yet apparently it is rich enough to classify regions. This is an important result, and I would be this forward in the writing.
Thank you for this comment. Initially we were worried about over-interpreting, but we added a sentence on lines 236-238 (page 10) of the results to highlight this more. We also agree that the result is quite striking initially, but after additional consideration it might not be that surprising (as also highlighted in Reviewer 2’s comment below: “In a sense…”). That the performance in classification is so high was also the motivation behind the technical focus on methods in our manuscript since we felt we needed to investigate this result carefully to validate and understand it.

In general, the abstract and much of the paper could be written to elucidate the neurobiological significance of the results. The paper is written form a very informatics centric approach and reading it suggests the work of a strong informatics analyst and the senior authors on the paper might add more context and neurobiological significance of the work. The paper would be considerably improved by stressing the implications and utility of the work rather than details of performance classification.

In conjunction with addressing the subsequent point, we have gone through our manuscript and added additional biological context and interpretation throughout. In our initial writing, we were wary of over-interpreting any individual result, so we thank the reviewer for providing the opportunity to include potential implications of this work on future research.

We’ve made line edits specifically for this point and/or the subsequent one in the following lines:

Abstract: 46-52, 69-70

Background: 93-98, 100-102

Results: 207-210, 222-225, 228-230, 275-276, 283-287, 383-388, 405-407, 439-440, 447-448

Conclusions: 521-542

As an immediate example, starting the article’s abstract with the sentence chosen is not very powerful with respect to the study. One of the value propositions of spatial transcriptomics is far from simply being a means to link data type, but rather to understand cell type specific expression in situ and its spatial distribution. This should be the spirit of opening the article in my opinion and not as a data link mechanism.

We could not agree more. The recent wave of single-cell technologies lacked information on the spatial origin of the cells they are sequencing. Spatially resolved methods are perfectly poised to fill this gap and allow us to answer questions about the spatial patterning of cell-type specific expression. This is powerful. We hoped to capture this link between single-cell and spatial methods (and in neuroscience other data too) by referring to spatial as a potential data link more generally, but we see now why we have undersold the potential value. We have updated this in the abstract on lines 46-52 (page 2) as well as in the introduction on lines 93-98 (page 4) (as also referenced in response to the previous point, as this is a specific example of the prior comment).

Lines 100-107. The enumeration of these opportunities. I would state more directly option 5 as what is being done with an explanatory justification of why.
We really appreciate this suggestion as we think it cleans up the introduction very nicely, thank you. We updated the text accordingly on lines 128-136 (page 5-6).

The methodology of the approach needs to be intuitively described early in the results section. That is, how are being performing the anatomic label recognition from the regularized fit? Not a technical description which can be delayed until the methods section but the reader needs to understand the computational nature of the approach before describing results. Specifically, line 161, what does it mean to classify a leaf ARA structure?
Thank you for pointing this out. We had hoped that the paragraph now on lines 177-197 (page 8) would serve this purpose, but we now see that the original version was insufficient and lacked an explanation of how AUROC was calculated from LASSO. Hopefully the new edits to this paragraph (some additions and moving some information from the next paragraph earlier to here) helps to clarify our approach without going into a technical, methods-like description.

Line 195-196. This low dimensional characterization for small numbers of PC’s accounting for substantial variance has also been observed by others and as the authors not likely arrives to highly spatially correlated expression patterns. However, these major axes of variance describe only the highest level of variation and must be seen in contrast to the result you demonstrate that leaf level structures in the ARA ontology can be identified. The conclusion I draw is that the PC approach offers little in the way of biological interpretation distinguishing small anatomic structures.
We completely agree with you that a low number of PCs across the whole dataset accounting for much variance would be in contrast to the result that leaf level structures could be identified. Applying PCA to the whole ABA dataset together does show that few PCs capture a good bit of the total variance (Supplementary Figure 3e). Though 200 PCs is still not enough to capture 80% of the variance in either dataset. We’ve added this observation to the manuscript lines 260-262 (page 11).

However, the PCA result originally reported in the manuscript was applied to the separate brain areas successively. So while on average, few PCs (2) captured 80% of the variance on a per brain area basis, these are not the same two PCs across the whole brain. We believe that this result is indeed compatible with our result that leaf structures of the ARA can be classified.

We apologize as we were obviously very unclear in reporting this result since reviewer 1 also pointed it out in points 7 and 21 above. As referenced above, we added clarifying text to the results and methods sections.

In a sense the ability of the ABA data to predict anatomic labels better than the spatial transcriptomic data may not be surprising. The spatial data is operating at a cell type specific label and may not have sufficient power to delineate anatomic at this coarser level. At the ABA level as the authors note this expression identifiability is helped by considerable auto-correlation in the spatial signal and smoothing due to partial volume effects of the smoothed grid data. The authors may offer potential insights into these differences and with respect to the anatomic interpretability of spatial transcriptomic data.

We agree that it actually might not be surprising that the ABA data predict the anatomic labels well, especially at the level we interact with the ABA data (after registration and smoothing).

Note that the ST data we use is not specific to single cells, since it merely captures all the mRNA in a 100-micron diameter spot (see methods). As is, there is not a mapping of mRNA to specific cells. However, there is a center-to-center distance of 200μm which could make the ST higher resolution, in some respects, relative to the ABA which has 200μm^3^ voxels that directly border each other. In other words, that there is more space between each sample in a slice in the ST could mean that the sampled expression is better resolved between each sample of the ST relative to the ABA.

In general, the reviewer prompts an interesting discussion point that we were happy to elaborate more on in our discussion on lines 507-512 (page 23). Given the lack of iterative registration as in the ABA, it is noteworthy that the classification performance of the ST is still quite good. The mix of expression across cell types detected by the ST is distinct enough across the brain regions to classify the ARA brain labels.

I see as somewhat problematic the use of the sagittal ABA data in this context, here the registration and hence the gridded data is less accurate and voxel level. This may not add that much to the analysis, in particular as the coronal data set was chosen to contain many of the most likely differentially expression genes and these will be the differentiating ones in the LASSO regression analysis.
We agree that in practice the sagittal ABA data ends up being mostly a wash, especially, as the reviewer points out, because the coronal set contains the genes most likely to show spatial patterning. When we have looked at a handful of specific genes assayed in both planes, the spatial patterning of sagittal expression does seem to be in conflict with the patterning in coronal samples. Further, as noted in the manuscript, the sagittal subset is extremely sparse consisting of zeros for 53.9% of the dataset. This partially motivated our interest in treating them as separate experiments in a part of our analysis. In combined analyses where sagittal data is included, we average the expression values for genes that are assayed in both planes or in multiple series. If anything, this likely makes the performance we observe worst, or more conservative, than if we had only looked at the coronal set. In the analyses separating out the sagittal subset as an independent ‘dataset,’ its distinctness falls out in the results and the sparsity is noted in interpreting the results. Finally, a positive feature of LASSO is, as the reviewer noted, that it is regularized. This L1 regularization drives most coefficients to zero, so many of these genes assayed only in the sagittal subset end up not contributing to our models anyway.

All in all this is an interesting paper that could be more impactful with a more implication focused delivery while relegating details of prediction tradeoffs and performance relegated to supplementary materials.

We completely agree that our manuscript ended up being a bit too technical and are very sympathetic to this view point. When originally putting the manuscript together and again while editing during revisions we found it difficult to balance the competing demands for explaining both the neuroscience and technical issues. As seen in Reviewer 1’s comments, there was a substantial need to discuss further technical issues. And as highlighted by Reviewer 2’s comments, there was also a substantial need to discuss the neurobiological significance further.

We were delighted to receive the combination of these two reviews with slightly dichotomous focuses, as we think they are representative of our intended audience for this manuscript. We think our careful evaluation of the replicability of ABA data, which occupies a central place in neuroscience, merits this, at times inharmonious, combination. In fact, we chose PLOS Biology specifically for its broad audience. During the revisions process, we took advantage of the lack of page limitations in PLOS to try to include more neuroscience-driven interpretations of our results in response to Reviewer 2’s comments, while still capturing technical details.
